# A novel Frizzled 7 antibody disrupts the Wnt pathway and inhibits Wilms tumor growth

**DOI:** 10.3389/fbioe.2025.1641137

**Published:** 2025-09-24

**Authors:** Einav Vax, Revital Caspi, Rachel Shukrun, Naomi Pode-Shakked, Oren Pleniceanu, Hana Golan, Michael Namestnikov, Michal Mark-Danieli, Ela Markovsky, Dekel D. Bar-Lev, Iris Barshack, Ronit Satchi-Fainaro, Orit Harari-Steinberg, Sanja Goldberg, Benjamin Dekel

**Affiliations:** ^1^ Pediatric Stem Cell Research Institute, Safra Children’s Hospital, Sheba Medical Center, Ramat Gan, Israel; ^2^ Gray Faculty of Medical and Health Sciences, Tel Aviv University, Tel Aviv, Israel; ^3^ Dana-Dwek Children’s Hospital, Tel Aviv, Israel; ^4^ Sagol Center for Regenerative Medicine, Tel Aviv University, Tel Aviv, Israel; ^5^ Department of Physiology and Pharmacology / Cancer Research and Nanomedicine, School of Medicine, Tel Aviv University, Tel Aviv, Israel; ^6^ Department of Pathology, Sheba Medical Center, Ramat Gan, Israel

**Keywords:** Frizzled 7, anti-Frizzled 7 antibody, Wilms tumor, cancer stem cells, Wnt/β-catenin signaling, targeted therapy, monoclonal antibody therapy, pediatric kidney cancer

## Abstract

Frizzled 7 (FZD7), a Wnt receptor that activates canonical Wnt/β-catenin signaling, has been implicated in multiple cancers, including Wilms tumor (WT), the most common pediatric kidney malignancy. We previously identified FZD7 as a marker of the WT cancer stem cell population and a potential therapeutic target. To evaluate this, we generated a panel of monoclonal anti-FZD7 antibodies using epitope mapping of the receptor and assessed their functional activity in primary WT cells and xenograft models. Among the panel, clone 288.1 induced significant cell death in primary Wilms tumor cells and inhibited cell proliferation and migration. This effect correlated with canonical Wnt signaling inhibition, a reduction in activated β-catenin and downregulation of Wnt/β-catenin target genes concomitant with diminished Wilms tumor cancer stem cell (CSC) markers. In vivo, treatment with anti-FZD7-288.1 significantly inhibited WT xenograft growth, resulting in reduced tumor volume. These findings demonstrate that FZD7 is a critical driver of Wilms tumor progression and support antibody-mediated FZD7 blockade as a promising therapeutic strategy.

## 1 Introduction

Wilms tumor (WT) is the most common renal tumor of infants and young children (Nakata et al., 2020), occurs in 1 of 10,000 children and account for 6% of childhood cancers; With improved combined therapy, WT survival rates have risen over the last 50 years to 85%–90%; however, for those who experience disease relapse or metastasis, even intensive regimens result in lower survival rates, approximately 50% (Spreafico et al., 2009; Geenen et al., 2007). Furthermore, survivors are at greater risk for a broad spectrum of adverse outcomes caused by chemotherapy and radiation therapy, such as late mortality and secondary cancers (Geenen et al., 2007; Robison et al., 2005). To minimize the adverse effects of chemotherapy and reduce relapsing disease, new therapeutic modalities are warranted. As previously reported by our work Pode-Shakked et al. (Pode-Shakked et al., 2013), first-line chemotherapies given within the IC50 restriction doses do not eradicate WT stem cells or WT-initiating cells (WT-SC/WT-IC) *in vitro*, while second-line drugs used for WT recurrence exert a stronger effect but do not eradicate all WT-ICs. These findings suggest that a higher dose is needed to achieve better outcomes. Importantly, Wnt pathway-related molecules such as FZD7, FZD2, and β-catenin were found to be overexpressed in our initial global gene expression analysis of Wilms tumor blastema propagated as human tumor xenografts. This overexpression was subsequently confirmed in additional WT samples. (Dekel et al., 2006).

Wnt signaling is a central regulatory pathway involved in controlling key functions of normal and malignant cells and has become an important target for cancer drug development in recent years. This pathway is vital for embryonic development, cell cycle regulation, inflammation and cancer (Clevers, 2006). Growing evidence indicates that Frizzled 7 (FZD7) plays a role in the development, progression, and metastasis of cancers. Upregulation of FZD7 was found to promote cancer through canonical activation of Wnt signaling pathway not only in Wilms tumor (WT), but also hepatocellular carcinoma (HCC), triple negative breast cancer (TNBC), cervical cancer, melanoma, acute lymphoblastic leukemia (ALL), renal cell carcinoma (RCC), colorectal cancer, esophageal cancer and gastric cancer (Tycko et al., 2007; Bengochea et al., 2008; Kim et al., 2008; Bilir et al., 2013; Yang et al., 2011; Deng et al., 2015a; Deng et al., 2015b; Sinnberg et al., 2011; Tiwary and Xu, 2016; Khan et al., 2007; Xu et al., 2016; Ueno et al., 2009; Tanaka et al., 1998; Kirikoshi et al., 2001). Furthermore, the inhibition of FZD7 expression and function in these cancer cells corresponds to the inhibition of Wnt/β-catenin signaling and suppression of tumor cell migration and growth (Xu et al., 2016; Ueno et al., 2009; Wei et al., 2011). Hypermethylation and downregulation of FZD7/Wnt inhibitors, such as secreted frizzled-related protein (sFLP), DKK (Dickkopf) and Wnt inhibitory factor 1 (WIF-1), are observed in several cancer types, further supporting the involvement of the FZD7 receptor in cancer growth (Suzuki et al., 2004; Willert et al., 2002; Dimitriadis et al., 2001). Mutations in Wnt signaling components downstream of FZD7 (such as APC) are observed in various tumors (King et al., 2012). However, the “Goldilocks model” of Wnt signaling previously suggested by Albuquerque and Smits et al. explains why FZD7/Wnt targeting should be explored. According to the model, the degradation complex is still partially functional even in cells with mutations; thus, upstream events at the receptor level can also be targeted to modify Wnt signaling in these cells (Albuquerque et al., 2002). In this work, we hypothesized that downstream dependence on receptor activation is required for successful antibody-mediated tumor eradication.

Moreover, previously we found that a commercial FZD7 antibody can induce cell death in cell cultures derived from primary WT (p-WT) cells, further suggesting that it is a valuable therapeutic target in WT cells (Pode-Shakked et al., 2011). Since FZD7 is located on the cell surface, it can be efficiently targeted by using antibodies, free or conjugated to a drug such as an antibody–drug conjugate (ADC). Vantictumab (OMP-18R5) was developed with the strategy of targeting a shared epitope among five of the ten Fzd receptors, including FZD7 (Gurney et al., 2012). In contrast, we investigated epitopes that minimally overlap frizzled family members and span varying areas of the receptor to achieve Frizzled 7 specificity and potentially enhance the clinical use of these proteins.

Herein, we present the generation of a novel specific anti-FZD7 monoclonal antibody that inhibits the Wnt pathway in Wilms tumor, reduces WT CSC markers resulting and diminished WT growth.

## 2 Results

### 2.1 Epitope mapping and preparation for antibody production

**SCHEME 1:**
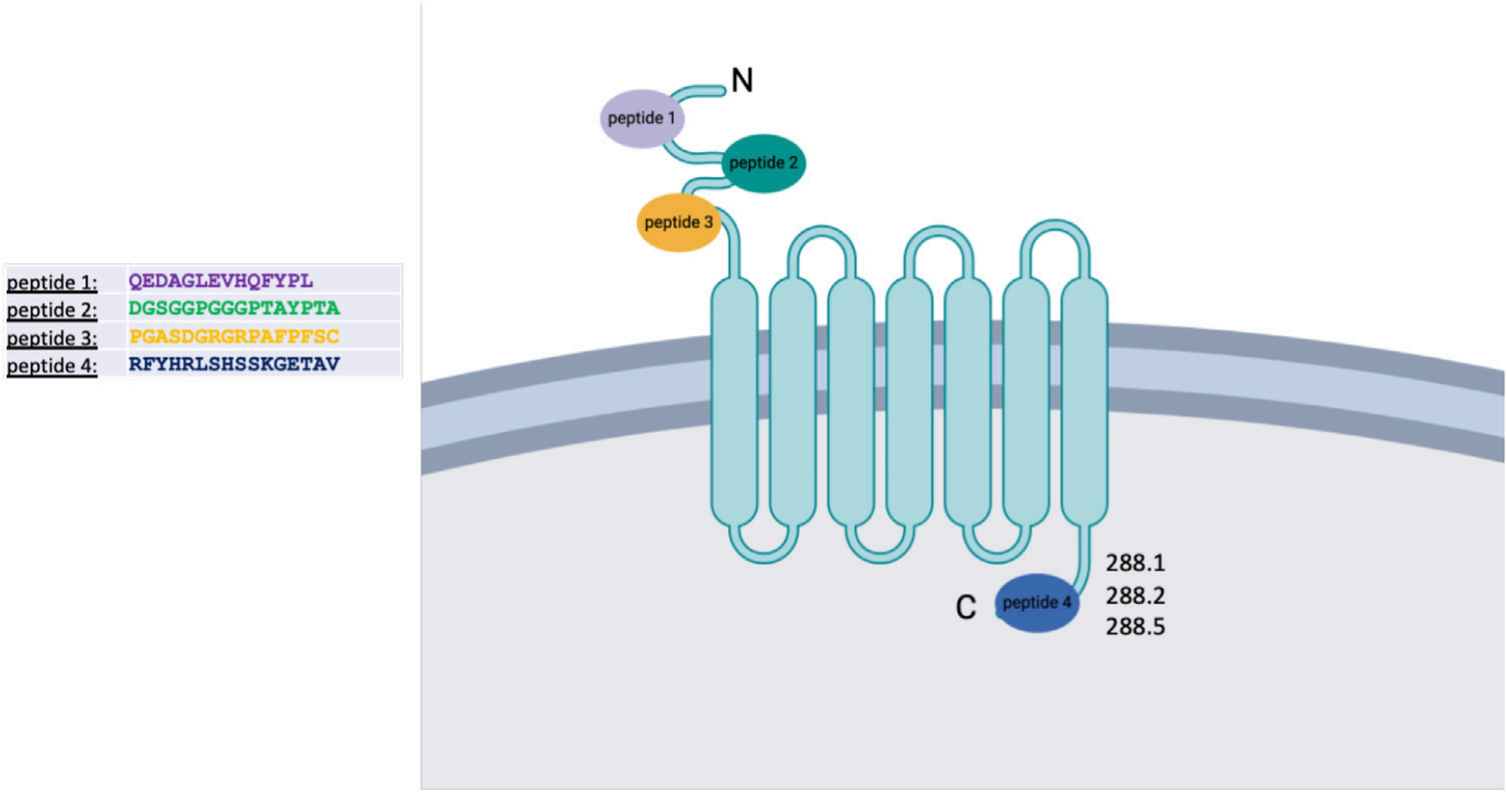
Schematic representation of the FZD7 protein structure and its localization within the cell membrane. Four peptides were selected for antibody generation; clones 288.1, 288.2, and 288.5 derived from peptide 4 were subsequently chosen for detailed analysis. Figure created with BioRender.com.

To identify potential unique and specific binding sites, we performed epitope mapping of the FZD7 protein. To ensure the selection of antibodies specific to the FZD7 receptor, four epitopes with minimal homology to other class frizzled (class F) G-protein-coupled receptors, consisting of 10 frizzled receptors (FZD (1–10)) and smoothened (SMO) (Schulte, 2010), were selected spanning throughout the FZD7 protein (the N-terminal region and C-terminal region), and peptides were generated accordingly (Scheme 1). Peptide 1 is a 14-amino acid (aa)-long sequence directed at a target antigen within the cysteine-rich domain (CRD) (Saldanha et al., 1998) and located at the N′ terminal region of the protein (aa76-aa89). Peptide 2, 16 aa long, also targets an antigen at the N′ terminal region (aa167-aa182). Peptide 3, 16 aa long, was directed at an antigen in close proximity to the transmembrane domain of the N′ terminal region of the protein (aa 195–aa 210). Finally, peptide 4, 16 aa long, was directed at an antigen located at the C′ terminal, intracellular region of the protein (aa559–aa574).

These peptides were injected into BALB/C mice (80 µg peptide/mouse) to generate specific antibodies according to standard protocols (Yokoyama, 2001). For each specific peptide, several antibodies were produced. Peptide matching was performed by ELISA to detect the specific target antigen of each antibody clone.

### 2.2 Functional screening of monoclonal antibody clones via the effect of supernatant on WT cells

**FIGURE 1:**
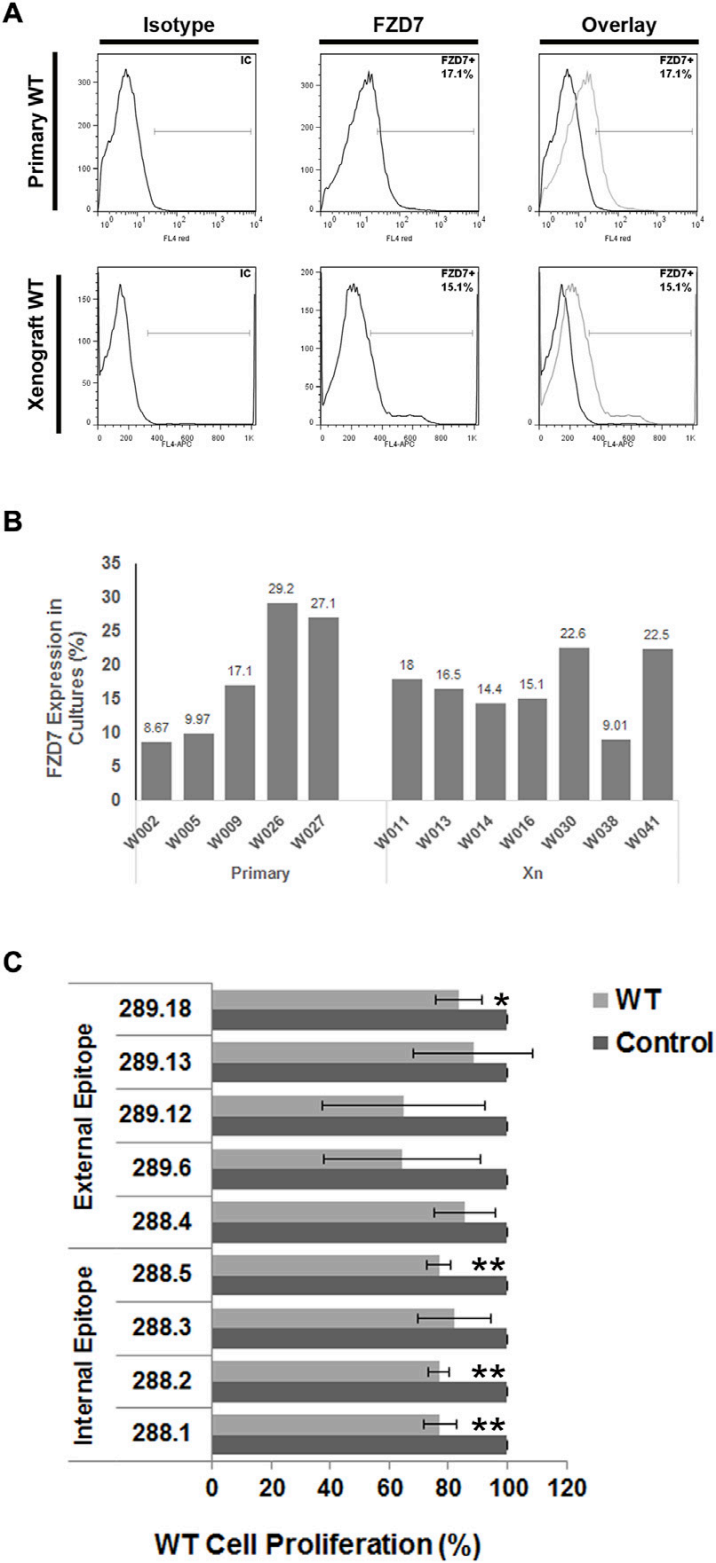
Binding of FZD7 antibody to Wilms tumor cells, expression analysis of different WT donors and effect on cell proliferation. **(A)** Percentage of Frizzle 7 (FZD7)-expressing cells in culture. A representative flow cytometry analysis is shown for primary Wilms tumor (WT)-derived cells (upper) and WT-xenograft derived cells (lower). **(B)** A summarizing bar graph of FZD7 expression in WT cells from different donors used for *in vitro* experiments. **(C)** Effect of secreted antibodies on WT cells: Relative percentage of viable cells following 48 h of treatment with the different clones. The spent medium served as a control. The numbers indicate the hybridoma clone from which the spent medium was derived; *p < 0.05, **p < 0.01. The results are shown as the mean ± SEM of at least three different experiments for each antibody.

To screen for potent monoclonal antibodies (mAbs), the effects of the supernatants of the hybridoma clones were first verified by ELISA on FZD7-expressing WT cells. Primary tumor cells obtained from various primary WT donors, along with cells derived from WT xenografts in immunodeficient mice, were analyzed for FZD7 expression levels using flow cytometry. FZD7 levels were monitored in the different tumors and remained steady throughout the experiments (Figures 1A,B).

Clones were analyzed by applying medium containing the secreted antibodies from the hybridoma cell culture to the assayed cells. After a 48-h treatment course, we analyzed the control and treated cells for viability.

Overall, 4 antibody-producing hybridomas generated from different immunizing peptides (Supplementary Table S1) demonstrated significantly reduced proliferation in WT cells (Figure 1C; *p < 0.05; **p < 0.01). Surprisingly, clones 288.1, 288.2, and 288.5 generated by immunization with the intracellular peptide 4 significantly inhibited WT cell proliferation. Two of those antibodies were chosen for further analysis: 288.1 and 288.5.

### 2.3 FZD7 protein–αFZD7 antibody-specific interaction

**FIGURE 2:**
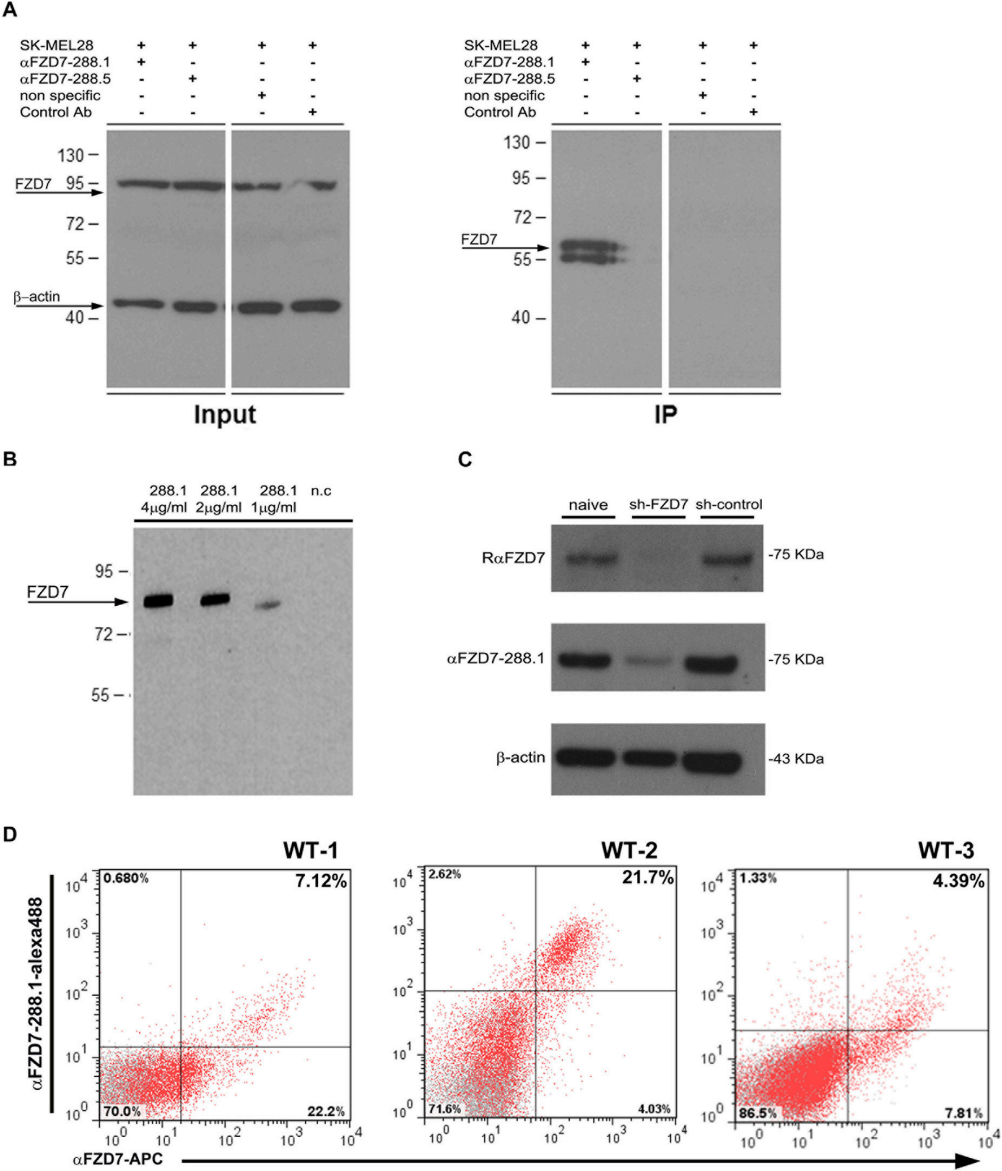
Binding characterization of FZD7 -288.1 in Immune Precipitation assays, Western blot analysis, FACS and Immunohistochemistry. **(A)** αFZD7-288.1 but not 288.5 immunoprecipitated the Frizzled 7 (FZD7) protein. A protein immunoprecipitation (IP) assay was used to validate the antibody-specific interaction with the FZD7 receptor. SK-MEL28 total cell lysates (FZD7 Input) and FZD7-bound fractions (FZD7 IP) were assayed via Western blot analysis. For detection, a commercial anti-FZD7 antibody (Millipore) was used. Nonspecific antibody and mouse IgG served as negative controls, and β-actin was used as a loading control. A representative blot of three separate experiments is shown. **(B)** Specific binding to the FZD7 receptor was examined in HEK293T cells overexpressing full-length FZD7. The cell extracts were probed with different concentrations of αFZD7-288.1. Specific binding of the Ab to FZD7-expressing cells was observed. **(C)** Probing of cell lysates from sh-FZD7-Wilms tumor (WT) cells with αFZD7-288.1 revealed the downregulation of FZD7, similar to the effect of a commercial αFZD7 antibody, demonstrating its specificity. β-Actin was used as a loading control. **(D)** Representative flow cytometry analysis of FZD7 expression in cultured WT cells stained with both αFZD7-288.1 and a rat anti-FZD7 APC-conjugated antibody. Cells stained positive for αFZD7-288.1 were mostly observed as a subpopulation within the cells detected by RαFZD7-APC. The data are presented in a dot plot graph showing FZD7 staining in red and isotype control staining (negative control) in gray.

Direct protein–antibody interactions were verified by a protein immunoprecipitation (P-IP) assay in SK-MEL28 cells, a human melanoma cell line that expresses an average of 15% FZD7 (Supplementary Figure S1). The results showed that P-IP of the FZD7 protein was successful when using αFZD7-288.1 but not 288.5 (Figure 2A). Since specific direct interactions were observed only for αFZD7-288.1, these interactions were further tested via functional *in vitro* experiments.

To test the specificity of αFZD7-288.1, we compared our generated αFZD7-288.1 antibody to a commercial anti-FZD7 Ab (Merck Millipore). Western blot (WB) analysis revealed a similar pattern for identifying the predicted 64 kDa protein, as well as its modified forms by phosphorylation, glycosylation, or both (Supplementary Figure S2). The target antigen of the selected antibody, αFZD7-288.1, was confirmed to be antigen 4 by blocking the antibody-receptor interaction following incubation with the immunizing peptide (Supplementary Figure S3).

Specificity of the combinations of the various concentrations of αFZD7-288.1 was evaluated in HEK293T cells overexpressing the full-length FZD7 (Figure 2B) and in WT cells in which FZD7 was knocked down by shRNA (Figure 2C). We found specific binding to the FZD7 receptor, as shown in 2B. Flow cytometry analysis of live WT cells stained for both αFZD7-288.1 and the αFZD7-APC antibody (R&D) revealed a range of overlapping subpopulations. Interestingly, variability was noted for both antibodies; however, cells stained positive for αFZD7-288.1 were mostly observed as a subpopulation within the cells detected by the commercial antibody (Figure 2D).

### 2.4 αFZD7-288.1 inhibits Wnt signaling in WT cells

**FIGURE 3:**
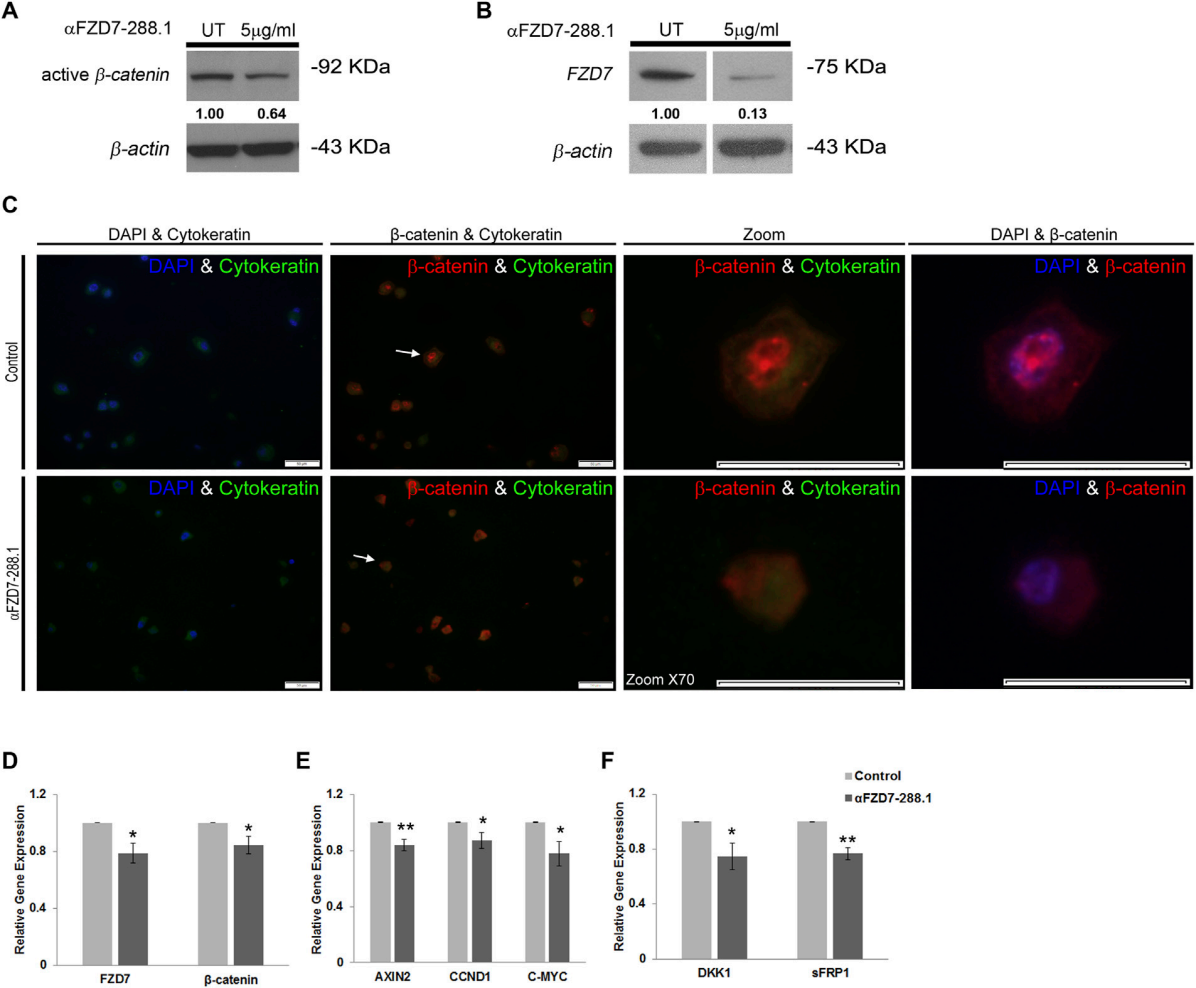
The αFZD7-288.1 antibody inhibits β-catenin translocating Wnt signaling in Wilms tumor cells. **(A)** Western blot (WB) analysis demonstrating the inhibition of active β-catenin and decreased levels of Fizzled 7 (FZD7). **(B)** following treatment with 5 μg/mL αFZD7-288.1 for 48 h in Wilms tumor (WT) cells. A nonspecific Ab was used as a negative control; β-actin was used as a loading control. **(C)** β-catenin immunofluorescence staining of untreated WT and αFZD7-288.1-treated cells: Double-staining with DAPI and cytokeratin (first panel) and with cytokeratin and β-catenin (second panel). β-catenin staining revealed strong expression and a greater percentage of cells with nuclear localization of β-catenin in the control untreated cells (scale bar represents 50 µM) X70 magnification of representative cells (indicated by arrows) from each group (third panel) and double-staining with DAPI and β-catenin (forth panel). **(D–F)** Real-time PCR gene expression analysis of WT cells: The expression of Wnt transducers [**(D)**; FZD7, β-catenin], Wnt pathway target genes [**(E)**; AXIN2, CCND1, MYC-C], and Wnt pathway inhibitors [**(F)**; DKK1, sFRP1] was also measured. The results showed reduced mRNA expression of Wnt pathway-related genes. The data are shown as the mean ± S.E.M. of 5 separate experiments; *p < 0.05; **p < 0.01.

Since αFZD7-288.1 was designed to target FZD7, a receptor for Wnt signaling, we aimed to examine its effect on this pathway to determine the underlying mechanism. WT cells were treated for 48 h with 5 μg/mL αFZD7-288.1, after which the protein levels of active β-catenin were measured via protein analysis. Active β-catenin and FZD7 protein levels were decreased in the cell lysates of treated WT cells (Figures 3A,B), suggesting that the whole signaling pathway is indeed downregulated following treatment with αFZD7-288.1. To further investigate the changes in β-catenin translocation following treatment, immunofluorescence staining of treated and untreated WT cells was performed. β-catenin is a key mediator of the canonical Wnt signaling pathway. In response to a Wnt stimulus or specific gene mutations, β-catenin is stabilized and translocated from the cytoplasm to the nucleus, where it binds to TCF/LEF-1 transcription factors to transactivate genes that drive tumor formation (Jamieson et al., 2014). Immunofluorescence staining demonstrated strong nuclear localization of β-catenin and weak cytoplasmic staining in the untreated cells, whereas the cytoplasmic localization of β-catenin was mostly observed in the treated WT cells (Figure 3C). These results suggest that αFZD7-288.1 treatment leads to inhibition of the Wnt/β-catenin pathway.

To explore the mechanistic impact of αFZD7-288.1-induced inhibition of Wnt signaling, we performed gene expression analysis of canonical Wnt pathway-related genes via real-time PCR. Our results demonstrate a decrease in the mRNA expression levels of *CTNNB1* (encodes β-catenin) and *FZD7*. Additionally, *C-MYC*, a known oncogene (He et al., 1998) and *AXIN2*, known to mediate tumor cell dedifferentiation and tissue-invasive activity (Yook et al., 2006) were also expressed at lower levels following the inhibition of Wnt signaling. Furthermore, *Cyclin D1 (CCND1)*, a Wnt target gene that was previously reported to be linked to cell cycle arrest, were correspondingly expressed at lower levels. This fact further links Wnt inhibition to tumor proliferation arrest (Tetsu and McCormick, 1999)*.* Finally, the Wnt pathway inhibitors *DKK1* (Chamorro et al., 2005) and *SFRP1* were also significantly decreased in WT cells (Figures 3D–F). As a control for specificity within the Wnt signaling pathway, αFZD7-288.5, which does not specifically interact with FZD7 (Figure 2A), was utilized. The mRNA levels of Wnt pathway-related genes remained unaffected by αFZD7-288.5 (Supplementary Figure S5).

### 2.5 Treatment with αFZD7-288.1 inhibits WT cell proliferation and induces cell death

**FIGURE 4:**
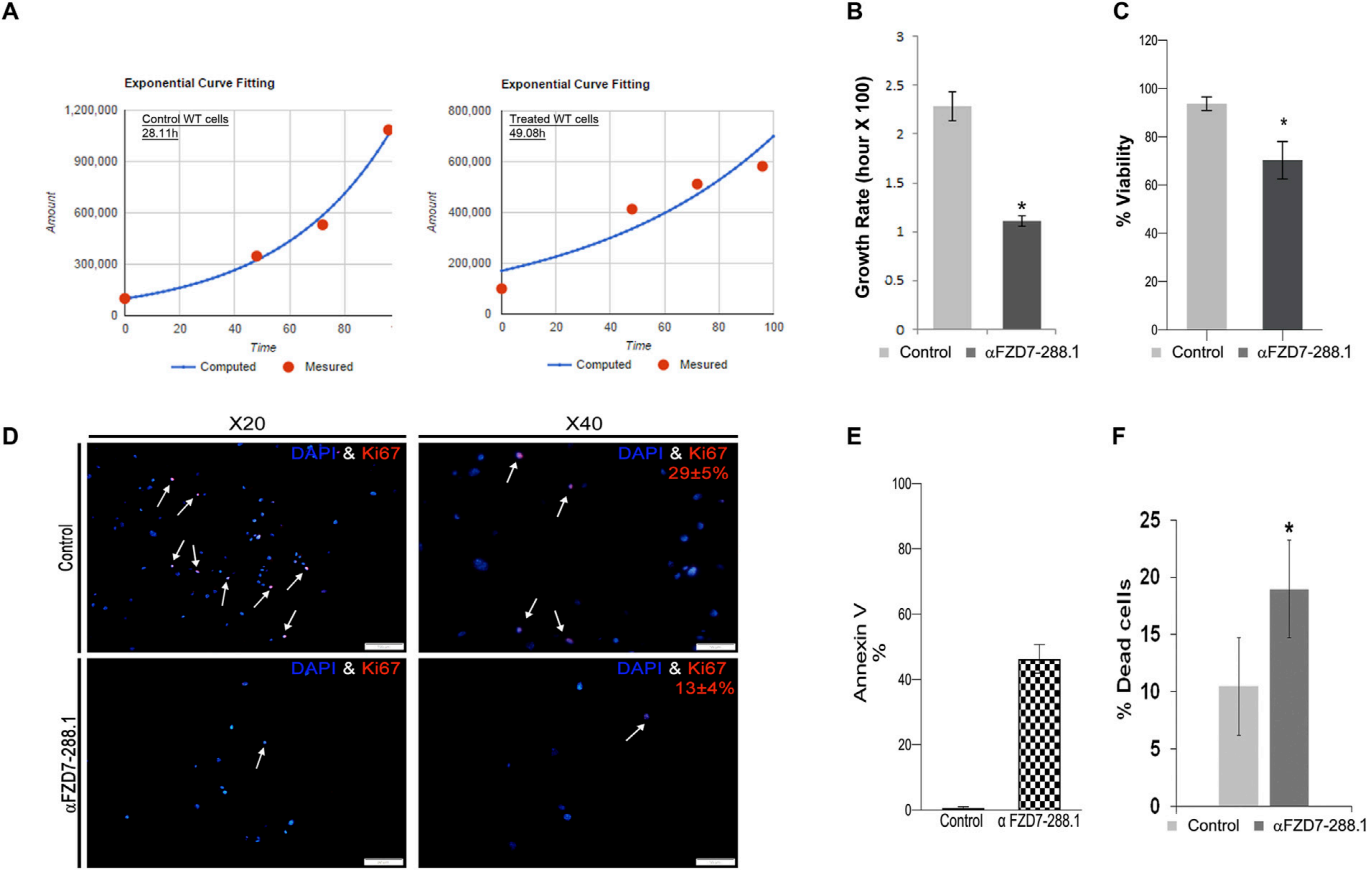
The αFZD7-288.1 antibody reduces proliferation in Wilms tumors cells and induces cell death. **(A)** Inhibition of the Wnt pathway by αFZD7-288.1 treatment induced changes in cell kinetics, as demonstrated by the longer doubling time of antibody treated Wilms tumor (WT) cells compared to control treated WT cells and **(B)** the significantly lower growth rate (defined as the number of cell divisions per day), *p < 0.05; n = 4. Reduced proliferation in cells treated with αFZD7-288.1 was also observed by MTS assay in **(C)** and **(D)** Quantification of Ki67 staining was performed by counting the number of Ki67-positive nuclei in five high-power fields (HPF) per condition, using representative regions from each sample. Reduced nuclear Ki67 expression (red, lower panels) compared to that in control untreated cells (magnification ×40 scale bars = 100 μm; magnification ×20 scale bars = 50 µm). **(E)** Induction of cell death in WT cells is shown in a representative analysis for annexin V staining, which demonstrated a marked increase in the percentage of apoptotic and preapoptotic cells. **(F)** Percentage of dead cells calculated using trypan blue staining of WT cells indicating significantly greater cell death in treated WT cells than in control cells; *p < 0.05; n = 4.

We further examined whether Wnt inhibition affects WT cell viability. Since the Wnt pathway is known to regulate cell proliferation and survival in various cancer types (Bilir et al., 2013; Sinnberg et al., 2011; King et al., 2012) and because of the highly proliferative nature of FZD7-expressing cells (Pode-Shakked et al., 2011), we first tested whether Wnt pathway inhibition via αFZD7-288.1 could lead to changes in cell proliferation. To evaluate proliferation, we calculated the cell growth rate (defined as the number of cell divisions per time unit). Doubling time was determined using a verified online calculator (R.V., 2006) based on measurements of cell numbers at 4 different time points (Figure 4A). Compared with untreated cells, αFZD7-288.1-treated WT cells had a significantly longer doubling time (approximately 49 h), which typically demonstrated a doubling time of 28 h and the significantly lower growth rate (defined as the number of cell divisions per day (Figure 4B). Reduced proliferation in cells treated with αFZD7-288.1 was also observed by MTS assay as shown in (Figure 4C). Further analysis of WT cell proliferation by immunofluorescence staining for Ki67 revealed reduced proliferation in treated cells, with only 13% Ki67-positive cells compared to 29% in control-treated cells (Figure 4D), consistent with their relatively decreased growth rate. Since Ab-mediated blockade of FZD7 was shown to induce cell death, we next examined whether treatment with our novel antibody, αFZD7-288.1, could result in cancer cell death. Flow cytometry analysis via annexin V staining of cancer cells treated with 5 μg/mL αFZD7-288.1 increased the percentage of preapoptotic and apoptotic cells (Figure 4E). Accordingly, using the trypan blue cell viability assay, we found that the percentage of cell death was significantly greater in the WT cells than in the controls (Figure 4F).

### 2.6 Treatment with αFZD7-288.1 inhibits WT cell migration

**FIGURE 5:**
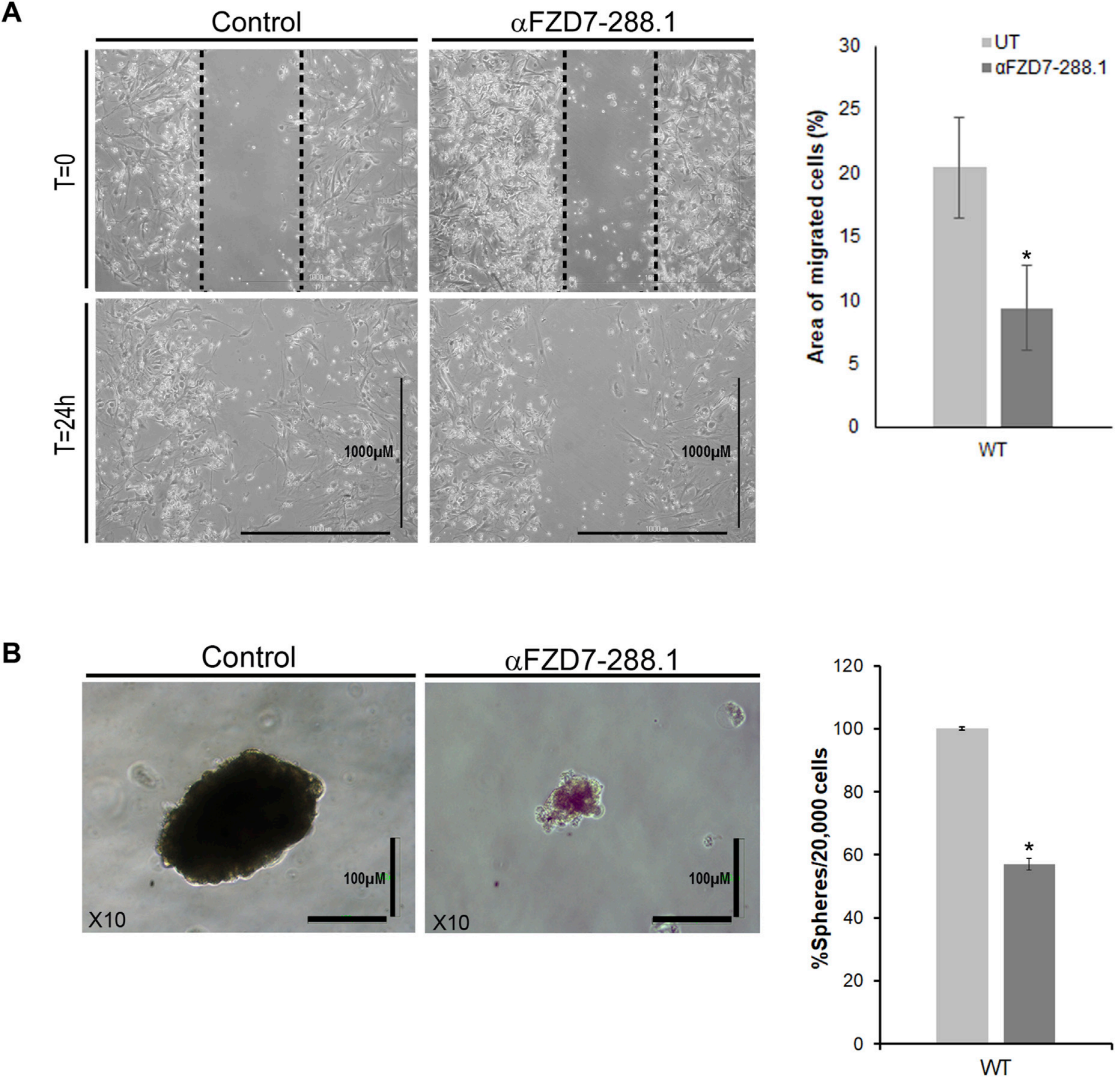
The migration and sphere formation of Wilms tumor cells is reduced after treatment with αFZD7-288.1 antibody. **(A)** Scratch assay demonstrating the effects of αFZD7-288.1 on the ability of cells to migrate through a scrape in the cell monolayer. Wilms tumor (WT)-treated cells demonstrated a lower migration capacity than control cells, as shown in the representative images. Calculation of the area of migrating cells (on the right) showed that the cells had a significantly reduced migration capacity following treatment; *p < 0.05; n = 3. **(B)** A sphere formation assay was performed. The number and size of the spheres formed by the treated cells were significantly lower than those formed by the untreated cells. The data are presented as the means ± SEMs of at least three experiments performed in triplicate (*p < 0.05) On the left, representative phase-contrast images of spheres formed by treated and untreated WT cells showing markedly fewer condensed spheres in the treated cells (scale bars = 100 μm, magnification ×10).

A common property of most cancers is their ability to migrate (Wirtz et al., 2011). Therefore, we assessed whether canonical Wnt pathway inhibition affects the migration potential of treated cells. We tested changes in the ability of WT cells to migrate through a scrape made in the cell monolayer in the serum-free medium to minimize proliferation and isolate migration effects. Inhibition of canonical Wnt signaling resulted in a reduced migratory capacity of the αFZD7-288.1-treated cells compared to the untreated cells (Figure 5A). An additional defining property of cancer stem cells (CSCs) is their ability to form cell spheres (Wang et al., 2018). Since FZD7 was previously shown to be a potential cancer stem cell marker in WT plants, we next asked whether treatment with αFZD7-288.1 affects this function. A sphere formation assay showed that a significantly lower number of spheres formed from treated cells than from control cells. Furthermore, the spheres that formed from the treated cells were smaller and less condensed (Figure 5B).

### 2.7 αFZD7-288.1 strongly co-localizes with NCAM1 in WT, and treatment with αFZD7-288.1 reduces WT CSC markers

**FIGURE 6:**
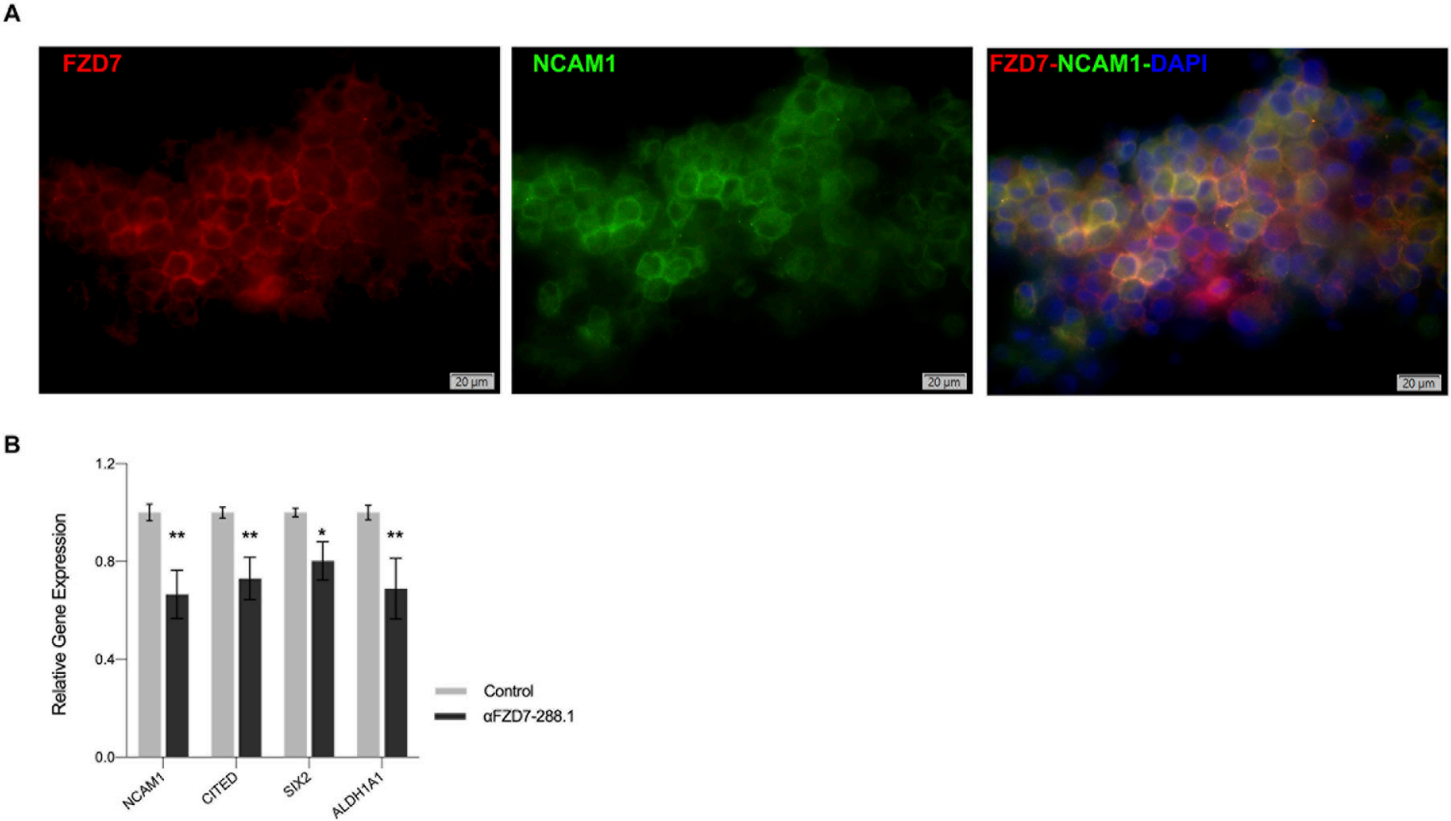
αFZD7-288.1 strongly co-localizes with NCAM1 in WT, and treatment with αFZD7-288.1 reduces WT CSC markers. **(A)** Immunofluorescence of staining of cultured WT cells using αFZD7-288.1 and NCAM1 and co-staining (αFZD7-red, αNCAM1 green and DAPI blue) microscopy revealed overlapping signals as a merged color (yellow), indicating membrane spatial proximity. Slides were imaged using Olympus IX83 fluorescent microscope and Olympus DP80 camera. Image processing was done using Olympus OlyVia software. **(B)** The expression of the Cancer stem cell marker genes NCAM1, CITED, SIX2 and ALDH1A1 decreased in the antibody αFZD7-288.1 treated WT derived cells (n = 3); *p < 0.05; **p < 0.01.

To determine the cellular compartment to which αFZD7-288.1 localizes, immunofluorescence (IF) staining was performed with antibodies against FZD7 and NCAM1, a WT CSC membrane-associated marker (Shukrun et al., 2014a). Cells were co-stained with fluorophore-conjugated antibodies against FZD7 (red) and NCAM1 (green), and merged images showed prominent yellow signals, indicating spatial membrane proximity (Figure 6A). Further, to evaluate the functional impact of FZD7 targeting, WT cells were treated with αFZD7-288.1 for 48 h. In line with previous reports identifying ALDH1^+^ and NCAM1^+^ cells as tumor-propagating populations with Wnt/β-catenin pathway activity and poor prognosis (Shukrun et al., 2014b; Raved et al., 2019), αFZD7-288.1 treatment led to significant downregulation of key CSC markers, including NCAM1, SIX2, CITED1, and ALDH1, as measured by qRT-PCR (Figure 6B). Notably, these markers overlap with populations resembling fetal nephron progenitors (SIX2^+^CITED1^+^) known to promote WT progression via ITGβ1–FAK–ERK signaling (Petrosyan et al., 2023). Together, these findings indicate that αFZD7-288.1 disrupts the stem-like compartment in WT, supporting a role for FZD7 in sustaining CSC properties. This underscores the embryonic plasticity of WT CSCs and highlights FZD7 as a potential therapeutic target to overcome resistance and limit tumor propagation.

### 2.8 Evaluation of the efficacy of αFZD7-288.1 in other cancer cells

Having shown the effect of αFZD7-288.1 on primary WT cells, specifically on CSC population, we explored whether αFZD7-288.1 has the potential to affect canonical Wnt signaling in other tumor cells. To achieve this goal, the melanoma SK-MEL28 and cervical cancer HeLa cell lines, previously linked to Wnt pathway aberrations (Deng et al., 2015a; Deng et al., 2015b; Tiwary and Xu, 2016; Spr et al., 2015; Anastas et al., 2014), were evaluated for FZD7 expression and treated with αFZD7-288.1. Treatment with αFZD7-288.1 inhibited the canonical Wnt pathway in both cell lines (Supplementary Figures S6, S7, respectively). These results support our hypothesis that αFZD7-288.1 may be a promising therapeutic option for a broad range of FZD7 canonical Wnt pathway-related tumors.

### 2.9 Inhibition of canonical Wnt signaling by αFZD7-288.1 inhibits Wilms tumor growth *in vivo*

Next, we sought to determine the effect of αFZD7-288.1 on WT growth via a xenograft model. In our previous study and in several other studies, treatment with the tubulin inhibitor paclitaxal (PTX) induced dramatic and favorable responses toward WT growth. Therefore, we compared the antitumor effect of αFZD7-288.1 to that of PTX (Markovsky et al., 2017; Ramanathan et al., 2000).

**FIGURE 7:**
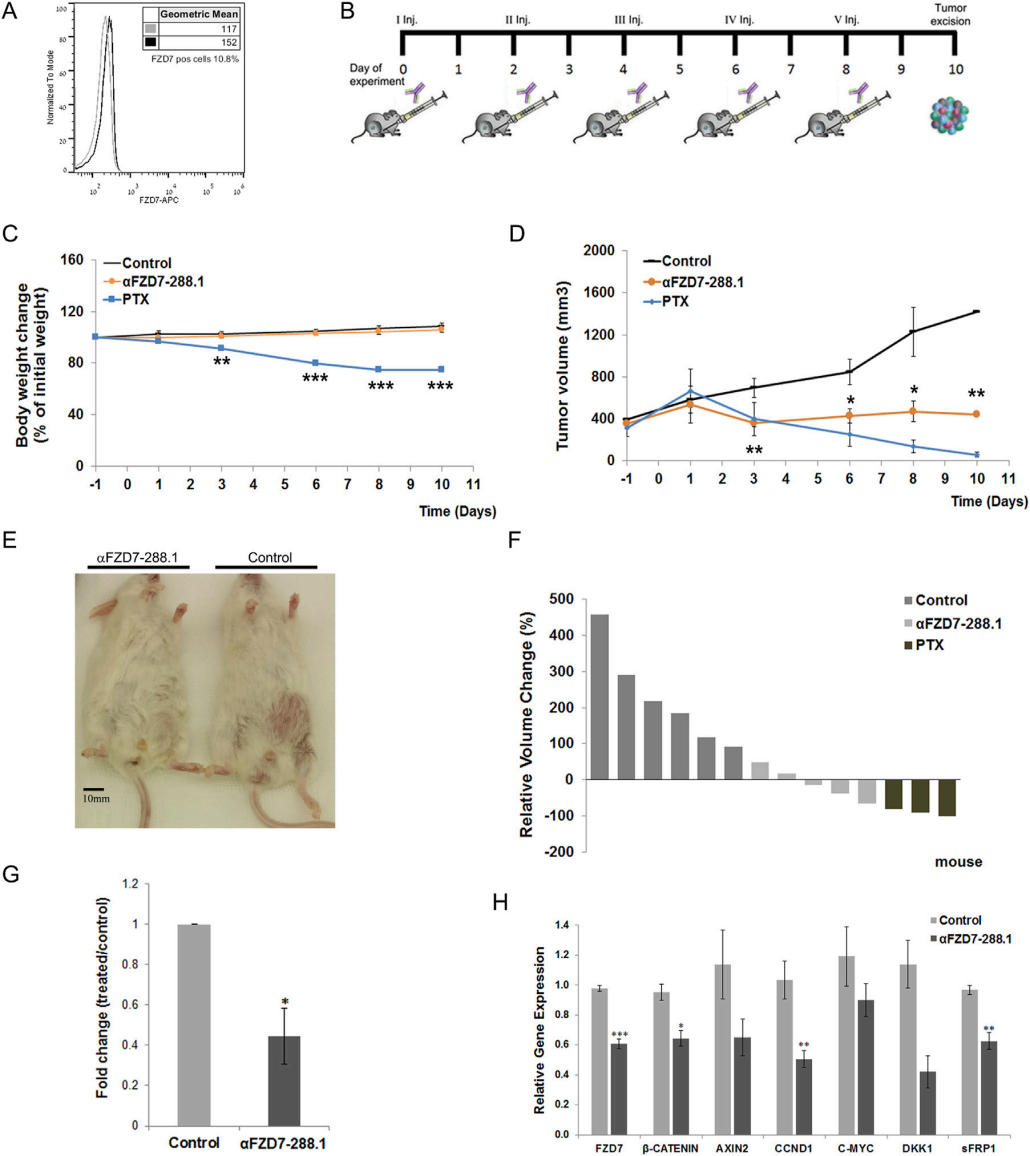
The antitumor activity of αFZD7-288.1 as demonstrated in changes in tumor volumes. **(A)** Flow cytometry histogram evaluating Frizzled 7 (FZD7) expression in Wilms tumor (WT) tissue for use *in vivo*. **(B)** Workflow diagram illustrating the *in vivo* experimental design. **(C)** Mouse weights during treatment. Paclitaxel (PTX)-treated mice suffered from toxic side effects and significant weight loss following treatment; **p < 0.01; ***p < 0.005. **(D)** Antitumor activity: Mice were treated intravenously with normal saline, αFZD7-288.1 (10 mg/kg) or paclitaxel (15 mg/kg). Tumor volume, presented in mm^3^, was assessed every 2–3 days using an external electronic caliper and calculated by the modified ellipsoid formula V = 0.52 × (length × width^2^). Like PTX-treated tumors, tumors treated with αFZD7-288.1 exhibited growth inhibition and even tumor shrinkage, while saline-treated tumors showed rapid tumor growth; *p < 0.05; **p < 0.01. **(E)** Representative images on day 10 of the experiment showing mice with large saline-treated tumors (right) and unnoticeable αFZD7-288.1-treated tumors (left). **(F)** Waterfall graph showing the changes in the individual tumor volumes with respect to the initial tumor size. **(G)** FZD7 expression in tumors treated with αFZD7-288.1 was significantly altered by 0.445-fold; *p < 0.05; n = 3 per group. **(H)** The expression of the canonical Wnt pathway genes FZD7, β-CATENIN, AXIN2, CCND1, C-MYC, DKK1, and sFRP1 decreased in the treated tumors (n = 4); *p < 0.05; **p < 0.01; ***p < 0.005.

The tumor injection protocol, tumor growth (volume) curves and body weight changes of each group are shown in Figure 7. Treatment with 10 mg/kg αFZD7-288.1 significantly inhibited tumor growth throughout the experiment starting after the second injection (p < 0.01). (Figure 7D). Three of the treated tumors were found to have a reduced volume compared to the initial tumor size (further in the text referred to as group A), while the other two tumors continued to grow but more slowly than did the control mice (further in the text referred to as group B) (Figures 7E,F). Like in the αFZD7-288.1-treated group, in the PTX-treated group, tumor growth was substantially inhibited after the second dose, followed by a decrease in tumor volume. Although PTX had a stronger and more rapid effect than did αFZD7-288.1 on all the tumors (n = 3; Figure 7F), the treated mice experienced weight loss, suggesting strong toxicity, which was not detected with the FZD antibody. This body weight loss of more than 20% following the fifth injection compelled us to stop the experiment.

Treatment of tumors larger than 500 mm^3^ with αFZD7-288.1 had similar results, with a delayed effect. A decrease in tumor volume was recorded in the treated tumors after the fifth injection (Supplementary Figure S8A). As mentioned above, since significant weight loss and death of three mice was recorded within the PTX-treated group, the experiment was terminated 10 days after the first inoculation (Supplementary Figure S8B).

Flow cytometry analysis of the tumors processed from the single-cell cohort demonstrated a significant decrease (fold change of 0.445) in the percentage of FZD7-expressing cells in the αFZD7-288.1-treated tumors compared to that in the control group (Figure 7G) and Supplementary Figure S8C. The expression of the canonical Wnt pathway genes of FZD7, β-CATENIN, AXIN2, CCND1, C-MYC, DKK1, and sFRP1 decreased in the treated tumors as measured by qRT-PCR (Figure 7H).

**FIGURE 8:**
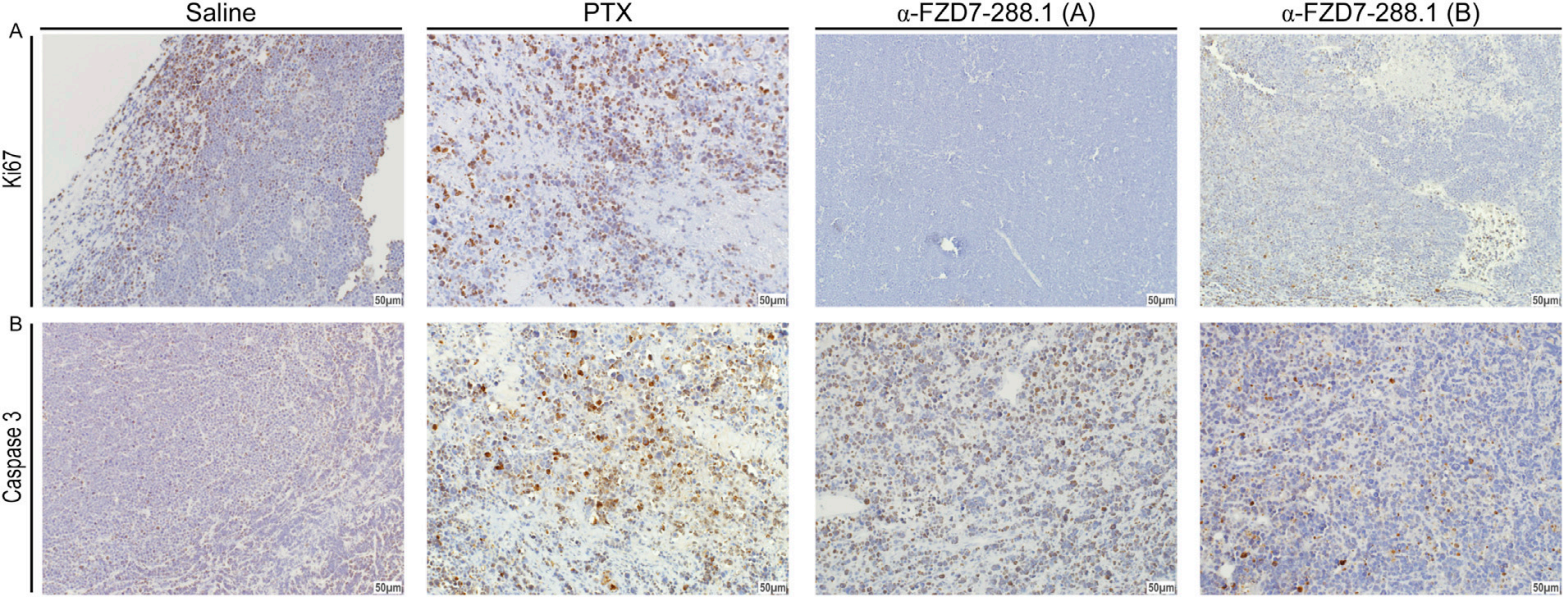
The antitumor activity of αFZD7-288.1 as demonstrated by proliferative markers in Immunohistochemical staining. **(A)** Immunohistochemical staining for Ki67 and Caspase3 in representative tumors from the different experimental groups; saline, paclitaxel (PTX) and αFZD7-288.1 (type A, representing reduced-volume tumors; and type B, representing tumors with growth inhibition compared to saline-treated tumors). The scale bars represent 50 μm at a magnification of ×20. Compared with those of saline- or PTX-treated tumors, αFZD7-288.1-treated tumors exhibited reduced proliferation. An increase in apoptosis was observed in αFZD7-288.1- and PTX-treated tumors **(B)**.

To further evaluate changes within the tumors, we performed immunohistological analysis of the tumors treated with saline, PTX and αFZD7-288.1. Two separate tumors representing tumors with reduced volumes (type A) and tumors with growth inhibition (type B) from the αFZD7-288.1-treated group were stained. Blocking Wnt signaling activity with αFZD7-288.1 resulted in reduced cell proliferation, as indicated by Ki67 staining, and induced cell death, as shown by extensive Caspase3 staining. Type A tumors exhibited minimal proliferation according to Ki67 staining and extensive Caspase3 staining, demonstrating increased apoptosis. These results coincide with the reduction in tumor volume observed during the *in vivo* experiment. Type B IHC staining revealed lower proliferation levels than did the control groups, and type B IHC staining was accompanied by lower apoptosis than was observed in type A tumors. This can account for the reduced response to αFZD7-288.1 treatment, as indicated by the inhibition of tumor growth. As expected, a similar effect was observed for the chemotherapy-treated tumors (Figures 8A,B).

## 3 Discussion

In this study, we generated a specific anti-Frizzled7 (FZD7) antibody that targets the Wnt pathway, reduces WT CSC markers and induces WT cell death. This study is a direct extension of our previous efforts to characterize WT-CSC/CIC (Dekel et al., 2006; Pode-Shakked et al., 2011; Pode-Shakked et al., 2009) and to demonstrate the effects of therapeutic targeting of putative WT-CSCs molecular markers with varying strategies (Pode-Shakked et al., 2013).

As a first step, a cohort of antibodies was produced that targeted different epitopes on the FZD7 receptor and viability/proliferation assays with WT cells were performed. Next, the specificity of the αFZD7 mAbs for the FZD7 receptor was tested. This analysis put forward the Ab termed αFZD7-288.1 generated by immunizing epitope 4 of the C′ terminus of the receptor, which was then tested successfully for Ab-receptor interactions via a protein immunoprecipitation (IP). We showed specific binding of the Ab to the tested tumor cells, in the context of live viable cells, as determined by flow cytometry, immuneprecipitation and WB analysis. We propose several explanations for the observed differences in the detected band sizes in input and pull-down fractions. Post-translational modifications (PTMs), such as phosphorylation or ubiquitination, may alter the protein’s molecular weight and mobility, with the input capturing modified/unmodified forms and the IP preferentially binding specific forms. Second, proteolytic cleavage may generate fragments seen pull down, with the IP detecting intact proteins or fragments. Further, antibody specificity may cause differences in epitope recognition, influenced by protein folding or interactions, with masking more likely in the input. The protein could also be part of a larger complex in the input, affecting its mobility, and dissociating during IP to appear at its expected size. Additionally, band patterns with the commercial antibody (Supplementary Figure S2) show similar patterns but varying intensities (e.g., 170, 90, 64 kDa), suggesting differences in IP performance. It is important to note that the 288.1 FZD7 antibody was selected for functional activity.

FZD7 was previously implicated as a potential cancer stem cell marker (Dekel et al., 2006; Pode-Shakked et al., 2011; Pode-Shakked et al., 2009), and considering that the current understanding implicates CSCs/CICs as the ones primarily responsible for tumor initiation and progression (Singh et al., 2003; van der Kooy and Weiss, 2000), we explored key elements of these characteristics. Herein, we showed that the application of the αFZD7-288.1 Ab can reduce cell propagation and induce cell death through targeting the Wnt pathway concomitant to abrogation of WT CSC markers. Our results indicate that αFZD7-288.1 prevents the accumulation of the active unphosphorylated β-catenin protein. Accordingly, we observed less active β-catenin in the nuclei. In fact, as we further investigated downstream gene transcription, we found that inhibition of the Wnt pathway by αFZD7-288.1 resulted in downregulation of its downstream targets. Notably, *CCND1*, a β-catenin target previously connected to cell cycle arrest, was significantly reduced, further connecting Wnt pathway inhibition to the reduced proliferation described above. *C-MYC*, a well-described oncogene and a target of the FZD7/Wnt pathway, was also repressed. Moreover, reduced expression of *β-catenin*, *FZD7* and the Wnt inhibitors *DKK1* and *sFRP1* was observed. This negative feedback may be the result of FZD7/Wnt pathway inhibition. Other key features of these FZD7-expressing cells include the formation of tumor spheres in low-adherence cultures and the migration of cells (Pode-Shakked et al., 2011). Focusing on these attributes, we went on to observe decreased migration capabilities following treatment, as well as impaired ability of the treated cells to form spheres. Although the inhibitory effects of the 288.1 antibody on WT cell proliferation and canonical Wnt signaling *in vitro* were modest (∼20%), even partial suppression of β-catenin activity can elicit meaningful biological outcomes—particularly in threshold-sensitive pathways such as Wnt. In our study, this moderate inhibition was consistently accompanied by reduced expression of stemness markers and impaired cell migration, highlighting the functional relevance of the antibody’s effect. Finally, our *in vivo* results in low-passage WT-Xn showed high antitumor activity of αFZD7-288.1, resulting in significant inhibition of tumor growth and tumor degradation. Moreover, analysis of the treated tumors revealed a significant decrease in the number of FZD7-expressing cells compared to that in the control group. This implies the possible elimination of FZD7-expressing cells from tumors and can account for the tumor volume reduction observed in three of the five treated tumors. The inhibition of the Wnt pathway gene expression module observed in the extracted tumors indicated that the remaining cells composing most of the tumor’s bulk were also affected by the treatment. This observation can be attributed to the inhibition of tumor growth following the inhibition of Wnt pathway activity, which is known to regulate cell proliferation (Bilir et al., 2013; Pode-Shakked et al., 2011; Vincan, 2004; Yang et al., 2011).Since no weight loss was measured in the αFZD7-288.1-treated mice, we did not reach the maximum tolerated dose of αFZD7-288.1; therefore, we hypothesize that the dose of αFZD7-288.1 should be increased further to improve its activity. We acknowledge the limitation of our *in vivo* study, particularly the small sample size and short observation period. To fully validate the therapeutic potential of the FZD7 antibody in Wilms tumor, additional *in vivo* experiments with larger cohorts and extended follow-up will be necessary. The mode of interaction of the αFZD7-288.1 Ab with FZD7 can be explained by several ways; One possibility is that the antibody is being internalized by the cells. Complex formation with FZD7 that is internalized as previously shown (Pode-Shakked et al., 2011). Alternatively, cell permeability or leaky membranes representing stressed cell state in tumor environments and stem cells or cancer cells, which are more prone to membrane dynamics, may allow access to intracellular compartments, even without specific transport mechanisms (Au et al., 2016). Additional explanation relates to FZD7 characteristics to undergo constant cycling between the plasma membrane and endosomes. During this process the cytoplasmic tail is transiently exposed in endocytic vesicles potentially binding FZD7 during its intracellular trafficking, affecting its recycling or degradation, thereby dampening Wnt signaling (Sun et al., 2016). In any event, rescue experiments that reactivate downstream Wnt signaling components can assist in directly testing reversal of the tumor-suppressive effects of αFZD7-288.1.

Since FZD7 is known to be involved in constant cycling between the membrane and cytoplasm and has a dynamic presence in the cell membrane (Pode-Shakked et al., 2011), the intracellular domain can potentially protrude from the membrane, affording attachment to the αFZD7-288.1 Ab. In this regard, a manuscript by Struewing et al. (Struewing et al., 2007) has been shown to cleave FZD7 loop 3, which may explain the instability of the FZD7 receptor. A manuscript published by Yang et al. (Yang et al., 2018) described a concerted movement in the helix VII and H8 regions of the FZD4 receptor at the proposed Dishevelled (DVL) binding site as the receptor cycled between a “closed” and a “bent” conformation during experiments. Since the αFZD7-288.1 target antigen is located only one amino acid downstream of the KTXXXW PDZ-binding motif of DVL, these findings may provide an explanation for the peptide mode of action. Moreover, a FZD7 peptide that was able to bind to the FZD7 receptor conventional C-terminal peptide-binding groove was previously reported (Wong et al., 2003). Regardless of the mode of interaction, our results suggest that αFZD7-288.1 is a promising therapeutic agent for treating WT cells and can be further implemented for other Wnt pathway-dependent tumors.

Standard chemotherapy drugs like vincristine, dactinomycin, doxorubicin, and cyclophosphamide remain the cornerstone of Wilms tumor treatment, especially for those with well-defined risk factors (Oostveen and Pritchard-Jones, 2019). However, targeted therapies like anti-FZD7 offer potential advantages in terms of specificity and reduced toxicity. The choice between these approaches depends on factors such as tumor stage, patient health, and the availability of targeted therapies. Combining traditional chemotherapy with targeted approaches could offer a synergistic benefit, potentially improving outcomes while minimizing side effects.

While our data support that αFZD7-288.1 exerts its effects through inhibition of Wnt signaling, demonstrating specificity is important next step. However, we recognize that such experiments would provide further mechanistic insight into the treatment’s specificity.

## 4 Conclusions

In summary, an anti-FZD7-288.1 novel antibody that specifically targets Fzd7 was successfully generated in this study. Monoclonal anti-FZD7- 288.1 enhanced cell death in primary Wilms tumor cells and inhibited tumor growth in *in vivo* xenograft studies. Mechanistically, we showed that this effect correlated with canonical Wnt signaling inhibition, a reduction in activated β-catenin, and downregulation of Wnt/β-catenin target genes. Our results suggest that the anti-FZD7-288.1 antibody is a possible therapeutic agent for Wilms tumor and additional Wnt-dependent tumors. Given that this antibody specifically targets FZD7 receptors, this specificity affords a beneficial safety profile for clinical use.

## 5 Experimental procedures

### 5.1 Antibody production

Monoclonal antibodies are raised against peptides corresponding to sequences specific to FZD7. Peptides used for antibody generation were synthesized by Sigma-Aldrich using solid-phase peptide synthesis (SPPS) with standard Fmoc (9-fluorenylmethoxycarbonyl) chemistry. Synthesis was performed on a resin support with stepwise addition of amino acids from the C-terminus to the N-terminus. Following chain assembly, peptides were cleaved from the resin and purified by reverse-phase high-performance liquid chromatography (RP-HPLC) to a purity of >80%. Peptide identity and purity were verified by analytical HPLC and mass spectrometry (MS). For immunization, the peptides were conjugated to KLH and injected into BALB/C mice (RRID:IMSR_ENV:HSD-162) (80 µg peptide/mouse) to generate specific antibodies according to standard protocols (Yokoyama, 2001). Upon clone selection, the clones were stabilized, produced in a bioreactor, and purified on a protein A-agarose column using AktaPrime (Pharmacia).

### 5.2 Primary WT samples

The primary WT sample was obtained from the patient within 1 h of surgery. Informed consent was given by the legal guardians of the patients according to the Declaration of Helsinki. The tumors were designated W002-W041, 12 of which were used in this study (Supplementary Table S3).

### 5.3 *In vivo* xenograft formation

Animal experiments were performed in adherence to the National Institutes of Health Guide for the Care and Use of Laboratory Animals, with institutional Animal Care and Use Committee approval. In this work, 5- to 8-week-old female nonobese diabetic severe combined immunodeficient NOD/SCID (RRID:IMSR_HAR:170) mice were used. WT xenografts (WT-Xn) were generated from immunodeficient mice as previously described (Pode-Shakked et al., 2013; Dekel et al., 2006).

### 5.4 Cell cultures

1. Cell lines: All cell lines were obtained from ATCC and tested negative for *mycoplasma* contamination.SK-MEL28 cells (RRID:CVCL_0526) were cultured in EMEM (Sigma‒Aldrich), and HEK293 (BCRJ Cat# 0009, RRID:CVCL_0045) and HeLa (TKG Cat# TKG 0331, RRID:CVCL_0030) cells were cultured in Dulbecco’s modified Eagle medium (DMEM; Biological Industries). All media were supplemented with 10% fetal bovine serum (FBS; Invitrogen), 1% penicillin streptomycin (Pen-strep) 100 M, and 1% L-glutamine (both from Biological Industries).
2. Wilms tumors: Single-cell suspensions from primary WT (p-WT) or WT-Xn tissues were grown as adherent cultures for *in vitro* assays. The cells were cultured in IMDM (Biological Industries) supplemented with 10% FBS and the following growth factors: 50 ng/mL basic fibroblast growth factor (bFGF), 50 ng/mL epidermal growth factor (EGF) and 5 ng/mL stem cell factor (SCF; R&D Systems) in 300,000 cells on a 10 cm plate on conventional tissue culture plastic or according to specific assay demands on surface area and cell density.
3. For sphere formation, cells were plated on poly (2-hydroxyethylmethacrylate) (poly-HEMA; Sigma‒Aldrich)-precoated 6-well plates (Corning Life Sciences, Wilkes Barre, PA, United States) at a density of 20,000 cells/well in serum-free DMEM-F12 (Invitrogen) supplemented with 10 ng/mL bFGF and 20 ng/mL EGF (R&D Systems). The medium was changed twice a week. After 7 days, the number of spheres and sphere sizes were determined using Nikon Eclipse TS100 and Nikon Digital Sight cameras, and the means were calculated.
4. To assess cell growth, 4,000 cells were plated in triplicate and grown in 96-well plates. Cell proliferation was measured by using a CellTiter 96 Aqueous One Solution Cell Proliferation Assay (Promega) according to the manufacturer’s instructions, and the absorbance at 492 nm was determined using an Infinite F50 microplate reader (Tecan).
5. For the determination of the doubling time and growth rate, 100,000 cells were seeded in 6-well plates. The following day and every day thereafter, the cells were trypsinized and counted using a hemocytometer. Calculation of the doubling time and growth rate (defined as the number of cell divisions per time unit) was carried out using a verified online calculator (R.V., 2006), which quantifies the doubling time of cells based on measurements of cell numbers at least three time points.
6. To assess cell death, 300,000 cells were seeded on a 10 cm plate. Annexin V staining was carried out using an annexin V–APC apoptosis detection kit (eBioscience) according to the manufacturer’s instructions. In addition, the cells were stained for 7AAD. The cells were subsequently analyzed with a FACSCalibur (BD Pharmingen). The results were analyzed using FlowJo analysis software. The trypan blue exclusion assay, based on the principle that live cells possess intact cell membranes and therefore exclude trypan blue dye, was also used to assess the number of viable cells in a cell suspension (Strober, 2001).
7. Quantification of Ki67 staining was based on the quantification of the number of positively stained nuclei in high-power fields (HPFs) from different areas of the sample (mean ± SD).
8. For assessment of cell viability after treatment, 4,000 cells were seeded in triplicate and grown in 96-well plates overnight. The following day, the medium was changed and supplemented with control or antibody.Cell viability was measured using the CellTiter 96 Aqueous One Solution Cell Proliferation Assay (Promega, Madison, WI, United States) according to the manufacturer’s instructions. The absorbance at 492 nm was determined using an Infinite F50 microplate reader (Tecan). Each experiment was performed in triplicate, and three independent experiments were carried out.
9. For the migration assay, cells were seeded into tissue culture dish at a density sufficient to reach full confluency within 18–24 h and incubated at 37 °C with 5% CO_2_. Once a uniform monolayer formed, a straight scratch was made across the center of each well using a sterile 200 µL pipette tip, applying consistent pressure and angle. Detached cells were removed by washing 2–3 times with PBS. The medium was then replaced with serum-free medium to minimize proliferation and isolate migration effects. Treated and control cells were photographed immediately after the scratch was made and 24 h later using a Nikon Eclipse TS-100 microscope.

### 5.5 Cell treatments

1. For preliminary experiments, cells were cultured on 96-well plates at 4,000 cells/well. Twenty-four hours after seeding, the cells were treated for an additional 48 h with spent medium from the different hybridoma clones containing the secreted antibodies.
2. The optimal concentration of αFZD7-288.1 was determined by culturing 4,000 cells/well in 96-well plates. After 24 h, the cells were treated for 48 h with different concentrations of the Ab. The following concentrations of Ab were used: 0–50 μg/mL for the WT cells or 0–20 μg/mL for the SK-MEL28 cells (Supplementary Figure S4).
3. αFZD7-288.1 treatment: Unless otherwise stated, cells were seeded on a 10 cm plate at 300,000 cells/plate. Twenty-four hours after seeding, the cells were treated by supplementing the growth media with 5 μg/mL αFZD7-288.1 for an additional 48 h of incubation. The cells were subsequently harvested and evaluated for the effect of αFZD7-288.1.

### 5.6 Flow cytometry analysis of FZD7 expression

10^5^ cells were suspended in FACS buffer supplemented with 0.5% BSA (Sigma‒Aldrich) and 2 mM EDTA in Dulbecco’s phosphate-buffered saline (PBS) (both from Biological Industries). The cells were then blocked with a mixture of FcR blocking reagent (Miltenyi Biotec) and human serum (1:1). The cells were stained with an isotype control (R and D Systems Cat# IC006A, RRID:AB_357254) or a primary antibody against FZD7 (R and D Systems Cat# FAB1981A, RRID:AB_2232278). Cell viability was tested using 7AAD viability staining solution (eBioscience). Cell labeling was detected using a FACSCalibur (RRID:SCR_000401). FACS results were analyzed using FlowJo (RRID:SCR_008520) analysis software. Viable cells were defined by their forward scatter/side scatter (FSC/SSC) profiles and lack of 7AAD staining. For analysis of p-WT and WT-Xn, freshly removed tumors were dissociated into single-cell suspensions as previously described and immediately analyzed. Cultured cells were detached using nonenzymatic cell dissociation solution (Sigma‒Aldrich) before staining.

### 5.7 Protein analysis

1. Protein isolation Cultured cells were treated as described above. Total protein was extracted using lysis buffer (1% Triton-X100, 20 mM Tris-HCl, 120 mM NaCl) and a tablet of protease inhibitor (Complete Mini Protease Inhibitor Cocktail; Roche). The mixture was incubated for 1 h on ice and centrifuged at 16,100 × *g* for 20 min.
2. Western blot: Protein concentrations were determined using the BCA-200 Protein Assay Kit (Pierce, Rockford, IL). Proteins were separated by SDS‒polyacrylamide gel electrophoresis (PAGE), transferred to nitrocellulose membranes and incubated with primary antibodies overnight at 4 °C. Secondary peroxidase-conjugated antibodies (Jackson ImmunoResearch, West Grove, PA; Supplementary Table S4) were used. Peroxidase activity was detected by exposing the membrane to (RRID) RRID:AB_2565817. and exposing it to medical X-ray film (Fuji).
3. Immunoprecipitation assay: SK-MEL28 cells (1 × 10^7^) were harvested in 1% NP-40 lysis buffer. Total cell lysates were incubated overnight at 4 °C with agitation with 5 µg of the tested antibodies, mouse anti-IgG, or nonspecific Abs as negative controls. The mixture was incubated in the presence of Protein A beads (Millipore), and the resulting complexes were washed, denatured and eluted according to the manufacturer’s instructions.
4. The peptide block αFZD7-288.1 was first incubated with peptide four at 4 °C with agitation for 4 hours. HEK293T (DSMZ Cat# ACC-635, RRID:CVCL_0063) cells overexpressing FZD7 and WT cells with only endogenous FZD7 expression were harvested and lysed as previously described. WB analysis of FZD7-knockdown cells was performed with the αFZD7-288.1 complex blocked by peptide four.

### 5.8 FZD7 RNA knockdown by short hairpin RNA (shRNA)

A sequence verification Sigma Mission viral vector (pLKO.1-puro)-based short hairpin RNA library (five clones targeting different sequences in the coding regions of the FZD7 gene, denoted as sh8343, sh8344, sh8345, sh8346, and sh8347; Supplementary Table S5) against FZD7 (FZD7-shRNA) was purchased from Sigma‒Aldrich. For transfections, the calcium phosphate protocol was used on HEK293 (BCRJ Cat# 0009, RRID:CVCL_0045) cells. 1 × 10^6^ cells were co-transfected with 10 µg of shRNA plasmid DNA vector, 5 µg of the packaging vector psPAX2 (RRID:Addgene_12260), and 2.5 µg of the envelope vector pMD2.G (RRID:Addgene_12259). The transfection efficiency of this procedure was determined by qPCR and reached 60%–70% at 72 h. Viral supernatants were collected 72 h post-transfection, filtered through a 0.45 μm PES filter, and concentrated by ultracentrifugation at 25,000 rpm for 2 h at 4 °C or using PEG-it Virus Precipitation Solution (System Biosciences), according to the manufacturer’s instructions. Viral pellets were resuspended in PBS and stored at −80 °C until use. Lentiviral titers were determined by qPCR-based titration (Lenti-X qRT-PCR Titration Kit, Takara Bio) following the manufacturer’s protocol, and the multiplicity of infection (MOI) was calculated based on these titers. For infections, target cells were seeded at ∼50% confluence and infected in the presence of 8 μg/mL polybrene (Sigma-Aldrich). Infections were performed at an MOI of 5. Transduced cells were selected with 2 μg/mL puromycin for 5 days starting 48 h post-infection. As a negative control, cells were infected with a MISSION non-targeting pLKO.1-puro lentiviral control vector (RRID:Addgene_8453).

### 5.9 qPCR analysis of gene expression

Total RNA was isolated using TRIzol reagent (Life Technologies) according to the manufacturer’s instructions. cDNA was synthesized using a High Capacity cDNA reverse transcription kit (Applied Biosystems, ABI). qPCR was performed using the StepOnePlus Real-Time PCR System (ABI) and the specific TaqMan Gene Expression assays for the relevant genes in the presence of TaqMan Fast Universal PCR Master Mix (both from ABI). *hHPRT1* and *hGAPDH* were used as endogenous controls (Supplementary Table S6). The results were analyzed using StepOnePlus Real-time Software in the ΔΔCT method, which determines the fold change in gene expression relative to a comparator sample. Statistical analysis was performed using a non‐paired two‐tailed t‐test. A p value < 0.05 was considered to indicate statistical significance. (Livak and Schmittgen, 2001; Schmittgen and Livak, 2008).

### 5.10 Immunohistochemistry and immunofluorescence staining of paraffin-embedded tissues

The samples were formalin-fixed and paraffin-embedded. Sections were pretreated using OmniPrep solution (Zytomed Systems) and blocked using Cas‐Block solution (Invitrogen) according to the manufacturer’s protocol. After 1 h of incubation at room temperature (RT), the sections were incubated with primary antibodies (Supplementary Table S7). The samples were then washed and incubated with secondary antibodies.

1. For immunohistochemistry (IHC), sections were incubated with HRP-conjugated goat anti-mouse (Vector Laboratories Cat# MP-7452, RRID:AB_2744550) and goat anti-rabbit (Vector Laboratories Cat# MP-7451, RRID:AB_2631198) antibodies for 30 min at RT. Staining was detected using an ImmPACT DAB kit (Vector) according to the manufacturer’s protocol. Hematoxylin was used for counterstaining.
2. Immunofluorescence (IF) staining of tissues was performed with donkey anti-mouse Alexa555 or donkey anti-rabbit Alexa488 (Life Technologies) for 1 h (Supplementary Table S7). Mounting with DAPI was performed using a DAPI Fluoromount-G (Southern Biotech). Images were obtained using an Olympus BX51TF fluorescence microscope with an Olympus DP72 camera and cellSens standard software.
3. Hematoxylin and eosin staining: 5 μm sections of paraffin-embedded Xn tissues were mounted on super frost/plus glass and incubated at 60 °C for 40 min. After deparaffinization, the slides were incubated in Mayer’s hematoxylin solution (Sigma‒Aldrich) and 1% hydrochloric acid in 70% ethanol for 1 min. The slides were then incubated for 10 s in eosin (Sigma‒Aldrich). Images were produced using an Olympus BX51TF fluorescence microscope with an Olympus DP72 camera and cellSens standard software.
4. IF staining of cells: Cells were fixed with 4% PFA and permeabilized with 0.1% Triton X-100 in PBS at RT prior to blocking with Cas‐Block solution (Invitrogen). The following steps were performed as described above. Slides were imaged using Olymus IX83 fluorescent microscope and Olympus DP80 camera. Image processing was done using Olympus OlyVia (RRID:SCR_016167) software.
5. Giemsa staining: Cells were washed with PBS and then fixed with 1 mL/well 100% methanol for 5 min. The wells were left to dry for 2 h and then stained with Giemsa for 5 min. The wells were washed with running distilled water for 3 min and left overnight at RT for complete drying before images were obtained (Olympus BX51TF).

### 5.11 *In vivo* treatment of WT with αFZD7-288.1

#### 5.11.1 Generation of WT xenografts from Xn-derived cells

For this purpose, a single-cell suspension from WT-Xn tissue was obtained by mincing the samples in Iscove’s Modification of Dulbecco’s Medium (IMDM) supplemented with antibiotics (penicillin and streptomycin), followed by treatment with collagenase IV for 1 h at 37 °C. Enzymatically treated tissue was triturated using IMDM at twice the volume of the collagenase solution, after which the suspension was filtered (100 μm followed by 70 μm cell strainer) and washed twice with IMDM containing antibiotics. Erythrocytes were removed with RBC lysis buffer (Sigma‒Aldrich). WT-Xn-derived cells generated in this manner were used for *in vivo* experiments. Approximately 0.75 × 10^6^ cells were injected in 100 μL of 1:1 serum-free medium/Matrigel (BD Biosciences) subcutaneously into the lower left flank of 6- to 8-week-old NOD/SCID female mice.

#### 5.11.2 Treatment of WT xenografts with αFZD7-288.1 or paclitaxel

Approximately 4 weeks after cell injection, when palpable tumors reached a volume no greater than 450 nm^3^, the mice were randomized into three groups: saline-treated, paclitaxel (PTX)-treated (15 mg/kg), and αFZD7-288.1 (10 mg/kg) groups. The study was conducted in a blinded manner where mice were randomly assigned to treatment groups, and drug formulations were prepared and coded by an independent investigator. Each mouse bearing a palpable tumor received five injections via the tail vein every other day. Tumor size was calculated according to measurements obtained by an external caliper using the modified ellipsoid formula V = 0.52 × (length × width^2^). Tumor dimensions and mouse weights were measured every other day beginning 1 day before the first injection. All enrolled participants finished the study, i.e., there was no attrition. Inclusion criteria for mice patient-derived xenograft (PDX) models treated with the drug included successful engraftment of tumor tissue, stable tumor growth, and adequate health status for treatment. Exclusion criteria included signs of severe distress or illness and death, and any pre-existing conditions that could interfere with drug efficacy evaluation. The experiment was terminated following 20% weight loss in the PTX-treated group according to the Helsinki protocol guidelines. Following resection, the tumors were divided into three pieces. One piece was fixed overnight in 4% buffered PFA and embedded in paraffin for histological staining to determine the recapitulation of the parental tumor and changes in tumor characteristics following treatment. Another piece was snap frozen and kept in liquid nitrogen for RNA extraction. The third piece was dissociated, and the cell suspension was immediately analyzed by flow cytometry for FZD7 expression.

### 5.12 Statistical analysis

The results are expressed as the mean values ± SEMs, unless otherwise indicated. Significant differences in xenograft size, cell viability (MTS assay), gene expression (qPCR) and surface marker expression (flow cytometry) were evaluated using a non‐paired two‐tailed t‐test. *In vivo* experiment was a pilot study; a formal power calculation was not applied. Statistical differences in the *in vivo* experiments were determined using Student’s t-test in Excel software assuming unequal variances. For all the statistical analyses, the level of significance was set as *p < 0.05 and **p < 0.01**.**

### 5.13 Summary

The anti-Frizzled 7 288.1 antibody shows promise by inducing cell death, inhibiting proliferation, and suppressing Wnt signaling, demonstrating efficacy in Wilms tumor xenograft studies.

## Data Availability

The original contributions presented in the study are included in the article/Supplementary Material, further inquiries can be directed to the corresponding authors.

## Glossary

ADC: Antibody–drug conjugate
ALL: Acute lymphoblastic leukemia
APC: Adenomatous polyposis coli
bFGF: basic fibroblast growth factor
CCND1: Cyclin D1
CIC: Cancer-initiating cell
CRD: Cysteine-rich domain
CSCs: Cancer stem cells
CTNNB1: Catenin (cadherin-associated protein) beta 1/β-catenin
DKK: Dickkopf
DMEM: Dulbecco’s Modified Eagle Medium
DVL: Disheveled
EGF: Epidermal growth factor
FZD: Frizzled
FZD7: Frizzled7
HCC: Hepatocellular carcinoma
ICC: Immunocytochemistry
IF: Immunofluorescence
IHC: Immunohistochemistry
IMDM: Iscove’s Modified Dulbecco’s Medium
LEF1: Lymphoid enhancer binding factor-1
mAb: Monoclonal antibody
NCAM: Neural cell adhesion molecule
NOD/SCID: nonobese diabetic severe combined immunodeficient
P: Passage of cells either in culture or in mice
PDZ: Amalgam of Postsynaptic density protein 95, Disc-large tumor suppressor and Zonula occludens
Pen-strep: Penicillin Streptomycin
P-IP: Protein immunoprecipitation
PTX: Paclitaxel
p-WT: Primary WT
qRT‒PCR: Quantitative reverse transcription PCR
RCC: Renal cell carcinoma
sFRP: Secreted Frizzled-Related Protein
shRNA: Short hairpin RNA
SMO: Smoothhened
TCF4: T-cell factor-4
TNBC: Triple-negative breast cancer
WIF-1: Wnt Inhibitory Factor 1
Wnt: Wnt ligands
WT: Wilms tumor
WT1: Wilms tumor1 protein
WT-IC: Wilms tumor-initiating cells
WT-SC: Wilms tumor stem cells
WT-Xn: Wilms tumor xenografts
Xn: Xenograft
