# Supplementary figures and images for: A novel Frizzled 7 antibody disrupts the Wnt pathway and inhibits Wilms tumor growth
Source: Front Bioeng Biotechnol. 2025 Sep 24;13:1641137. doi: 10.3389/fbioe.2025.1641137 (PMC12504202; doi:10.3389/fbioe.2025.1641137)

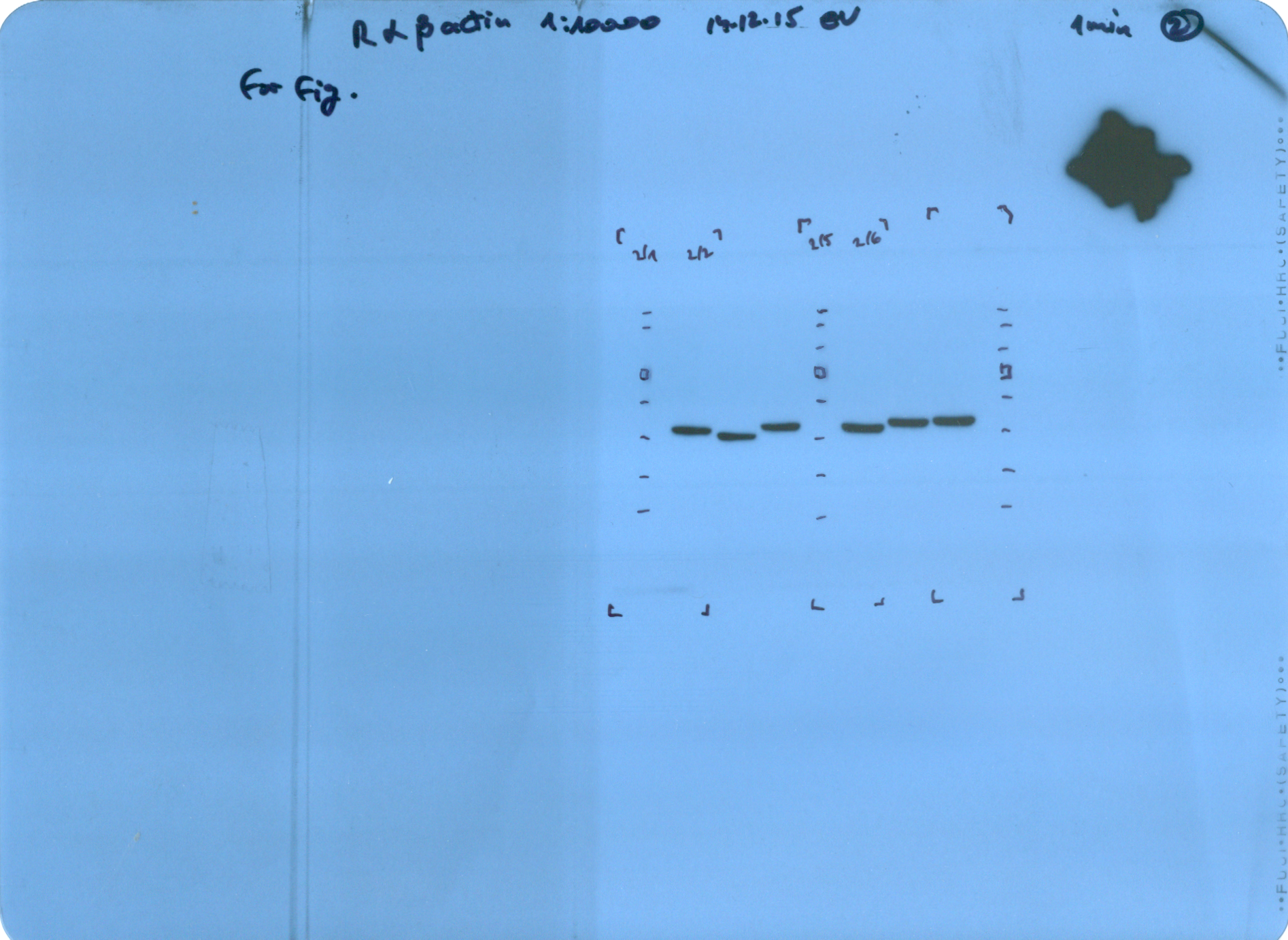

Supplement: Supplementary file 1 [file Image6.tif]

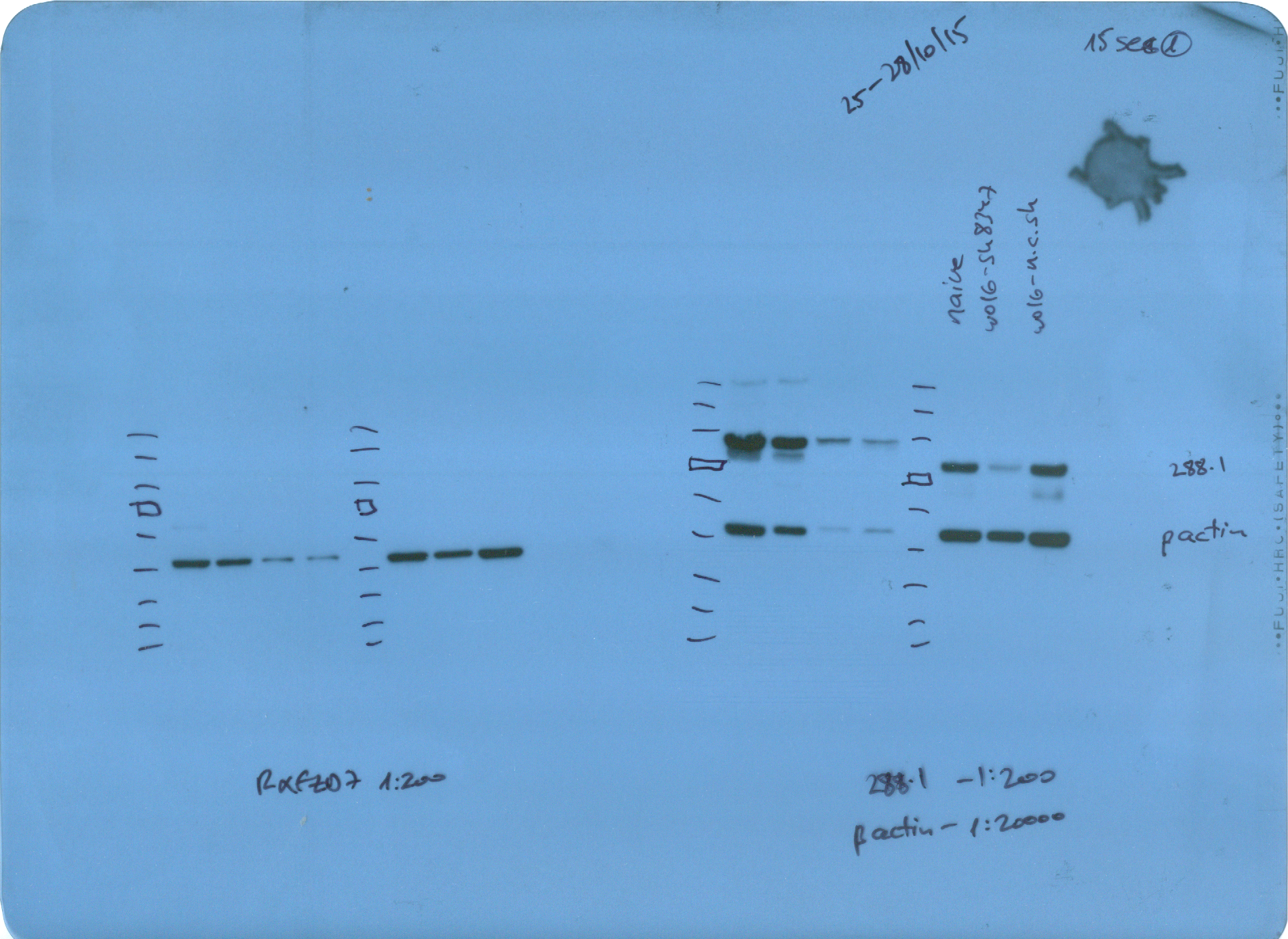

Supplement: Supplementary file 3 [file Image3.tif]

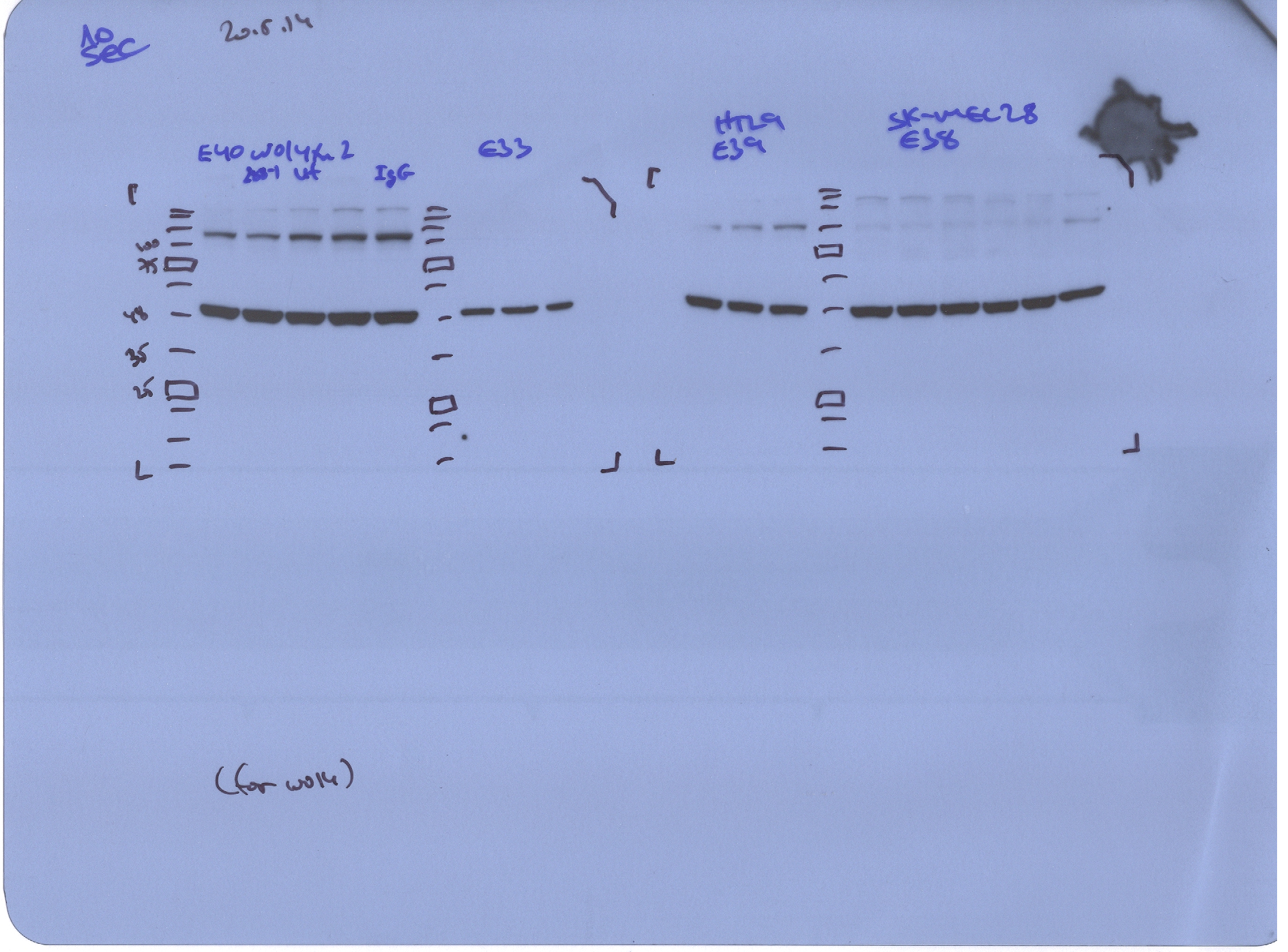

Supplement: Supplementary file 4 [file Image4.tif]

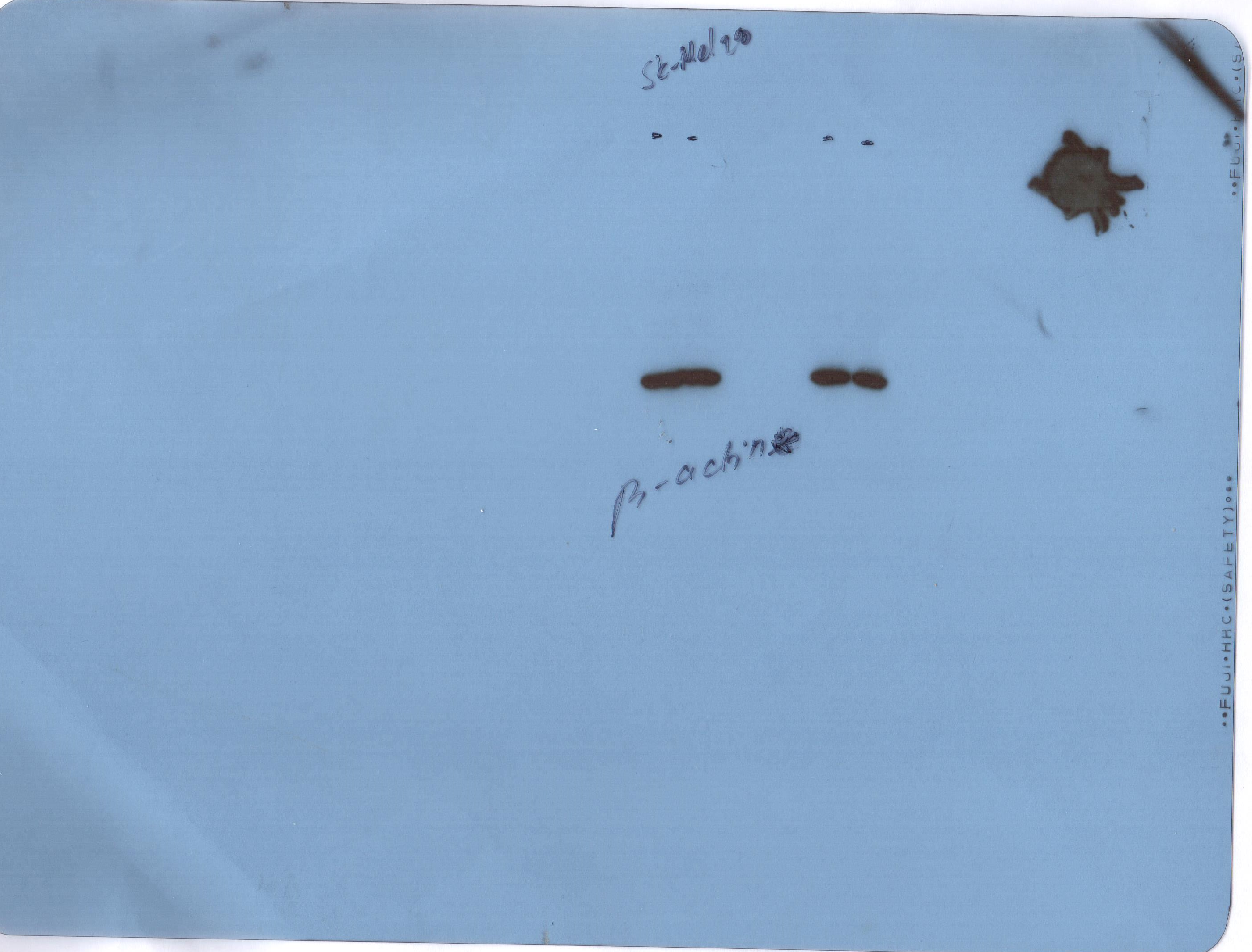

Supplement: Supplementary file 5 [file Image9.tif]

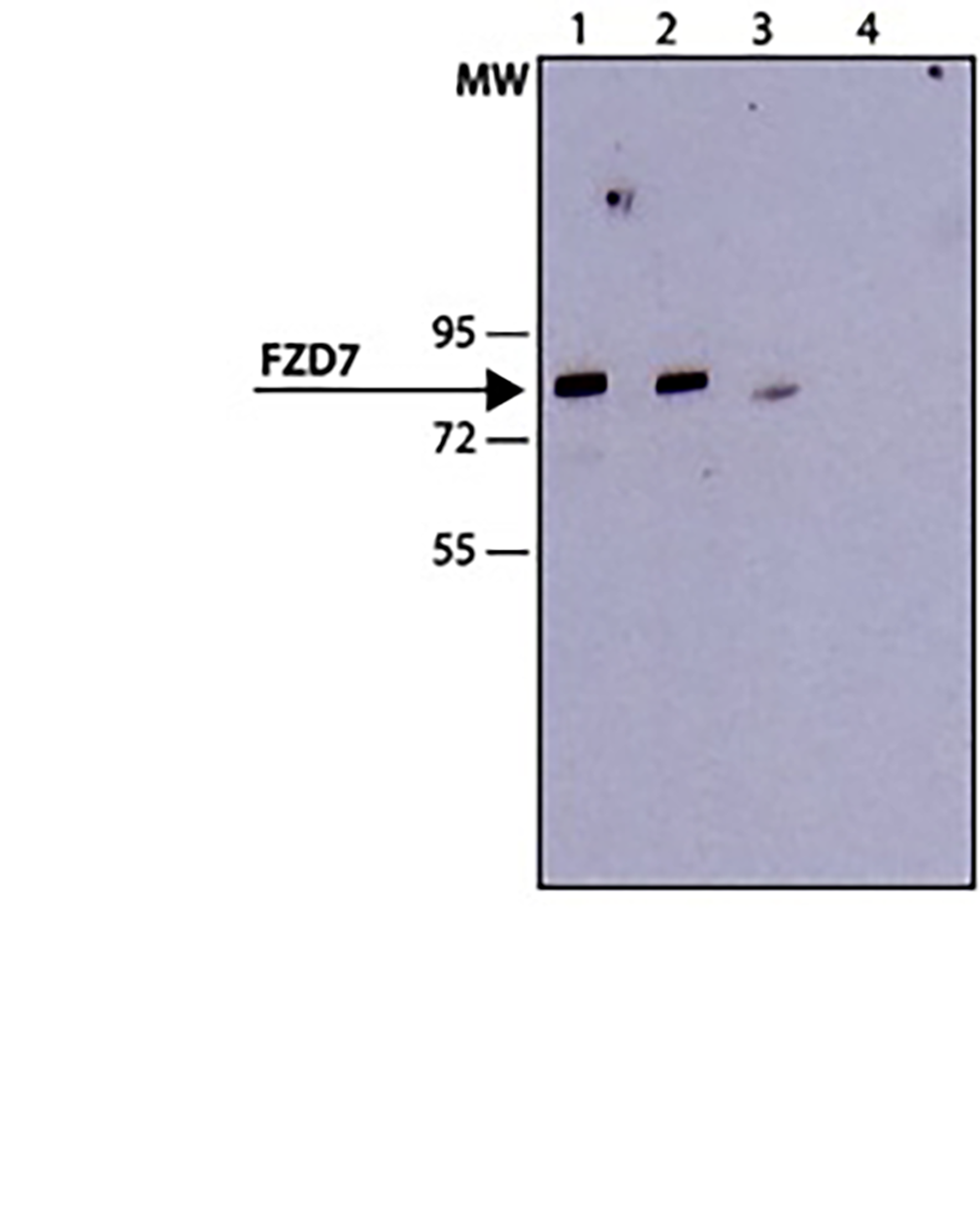

Supplement: Supplementary file 6 [file Image2.tif]

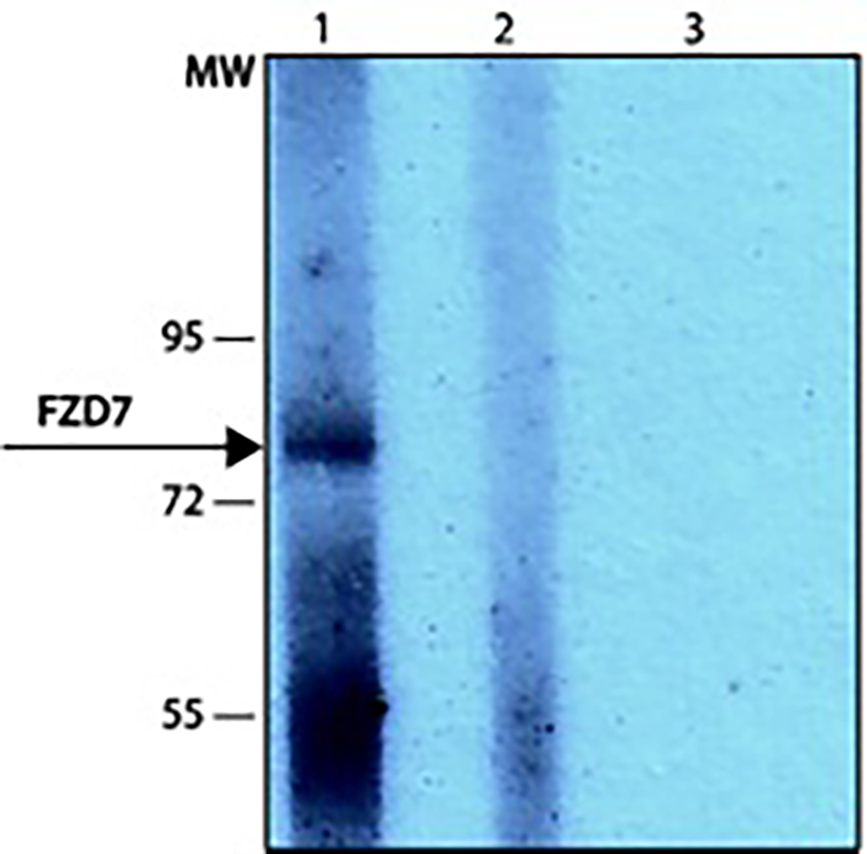

Supplement: Supplementary file 7 [file Image11.tif]

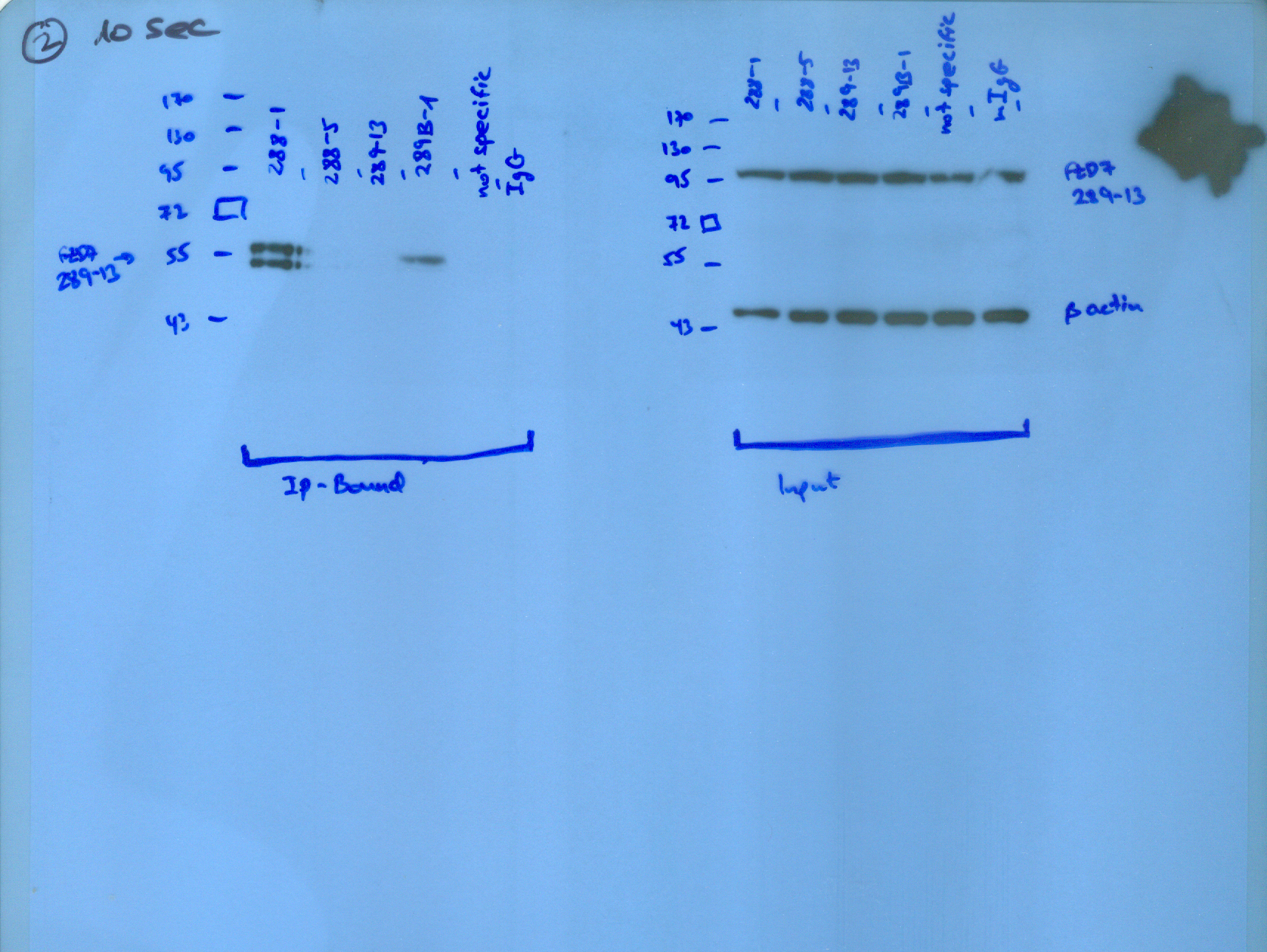

Supplement: Supplementary file 8 [file Image1.tif]

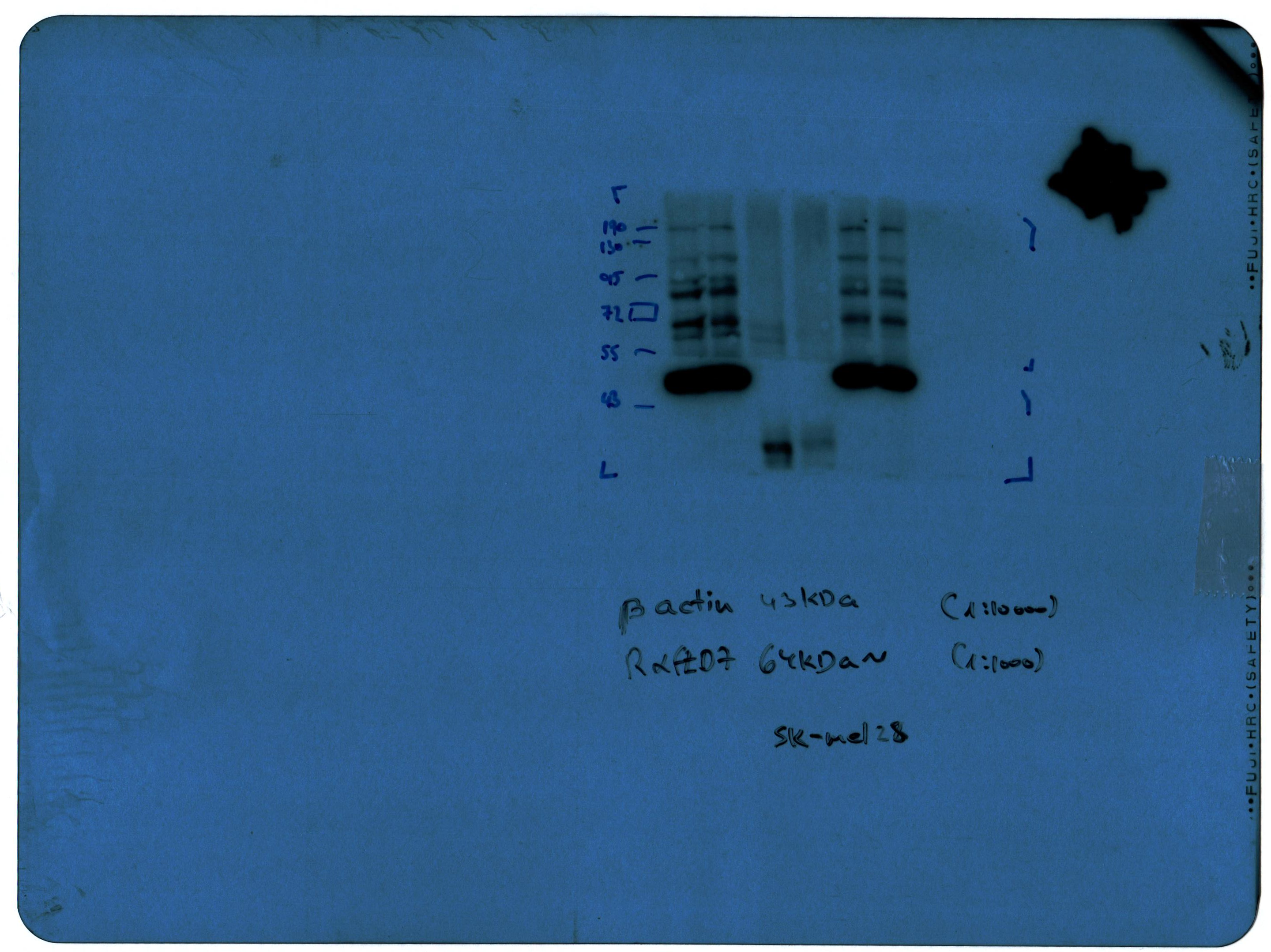

Supplement: Supplementary file 9 [file Image8.tiff]

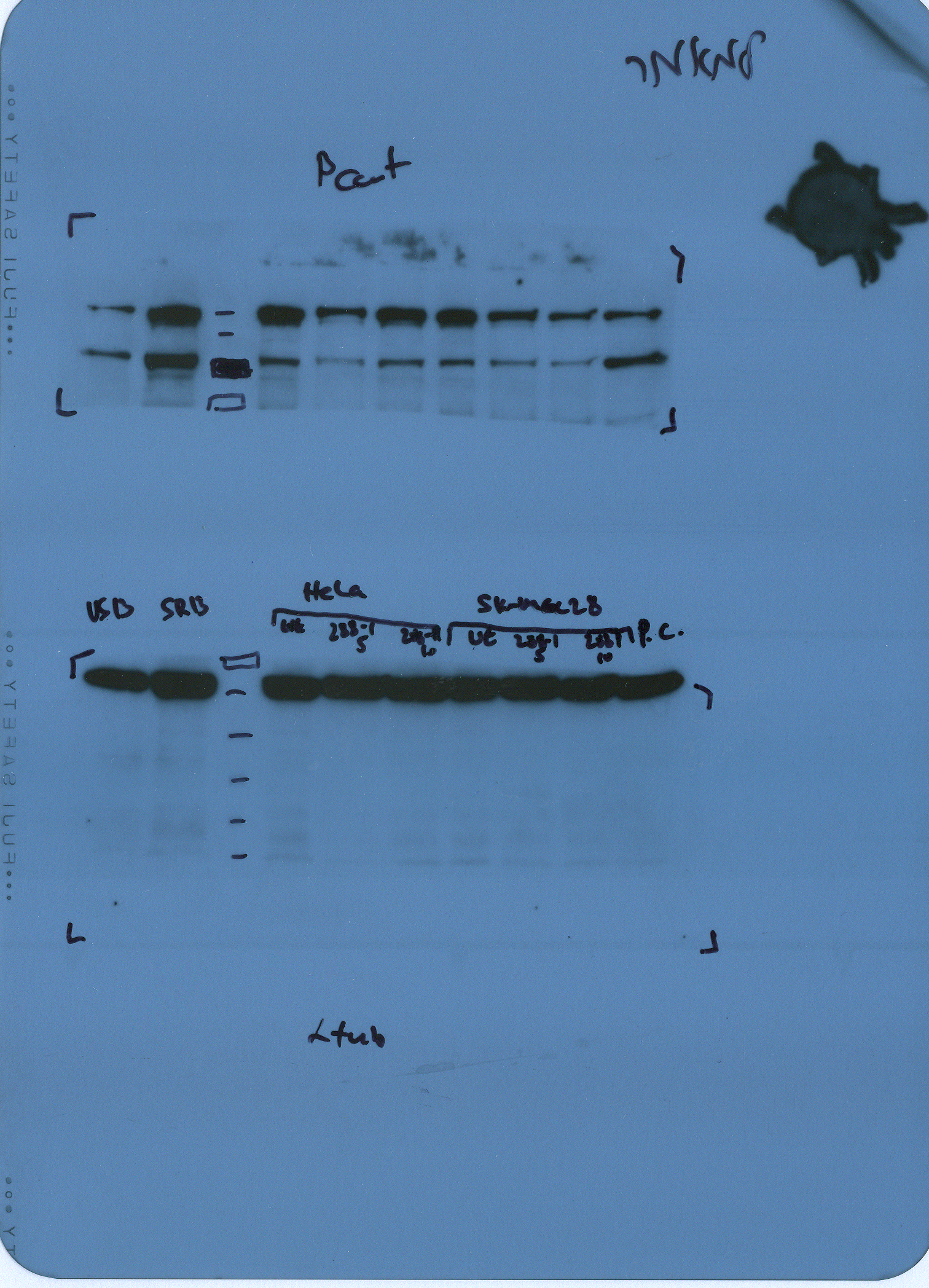

Supplement: Supplementary file 10 [file Image10.tif]

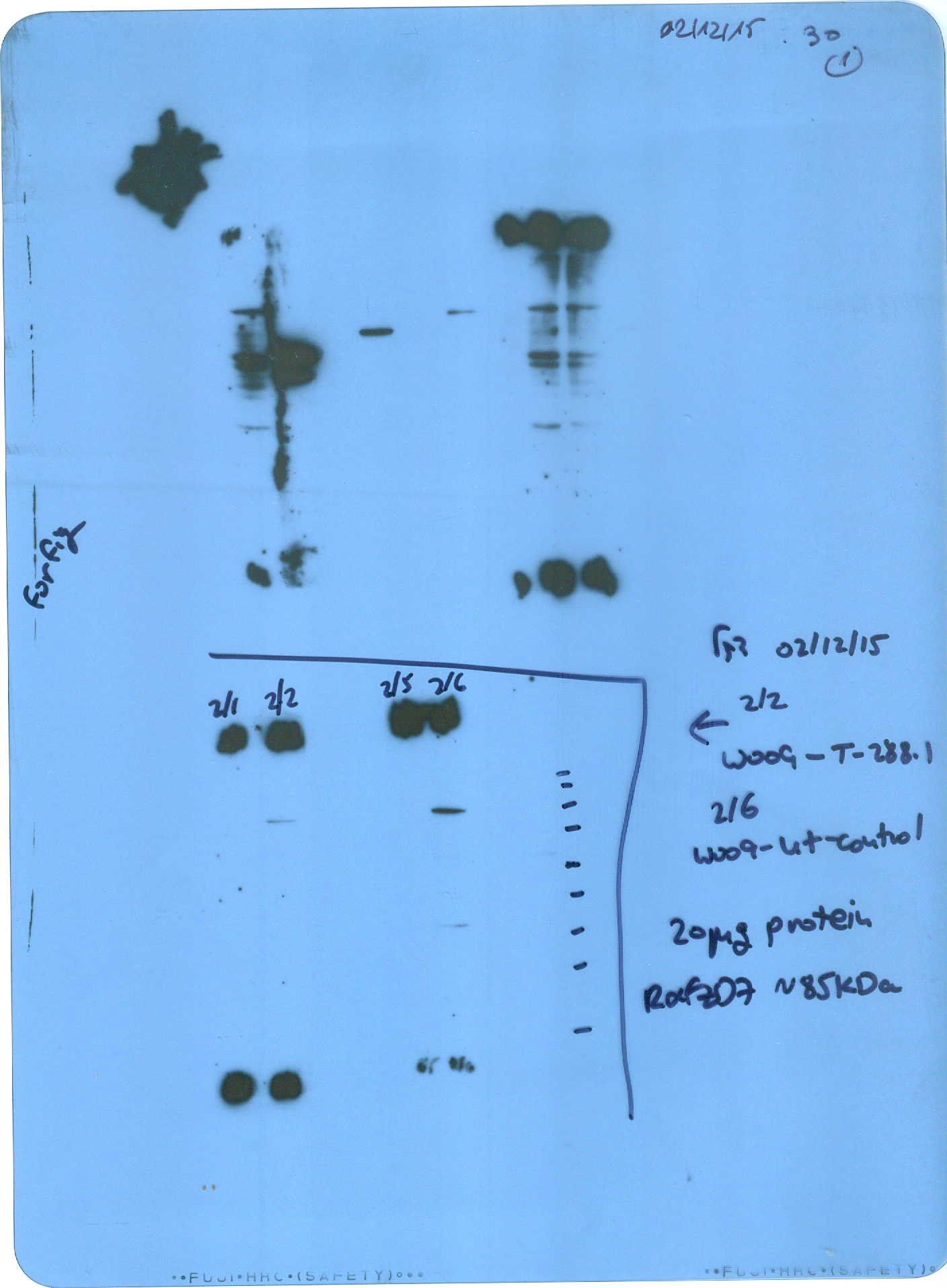

Supplement: Supplementary file 11 [file Image5.tif]

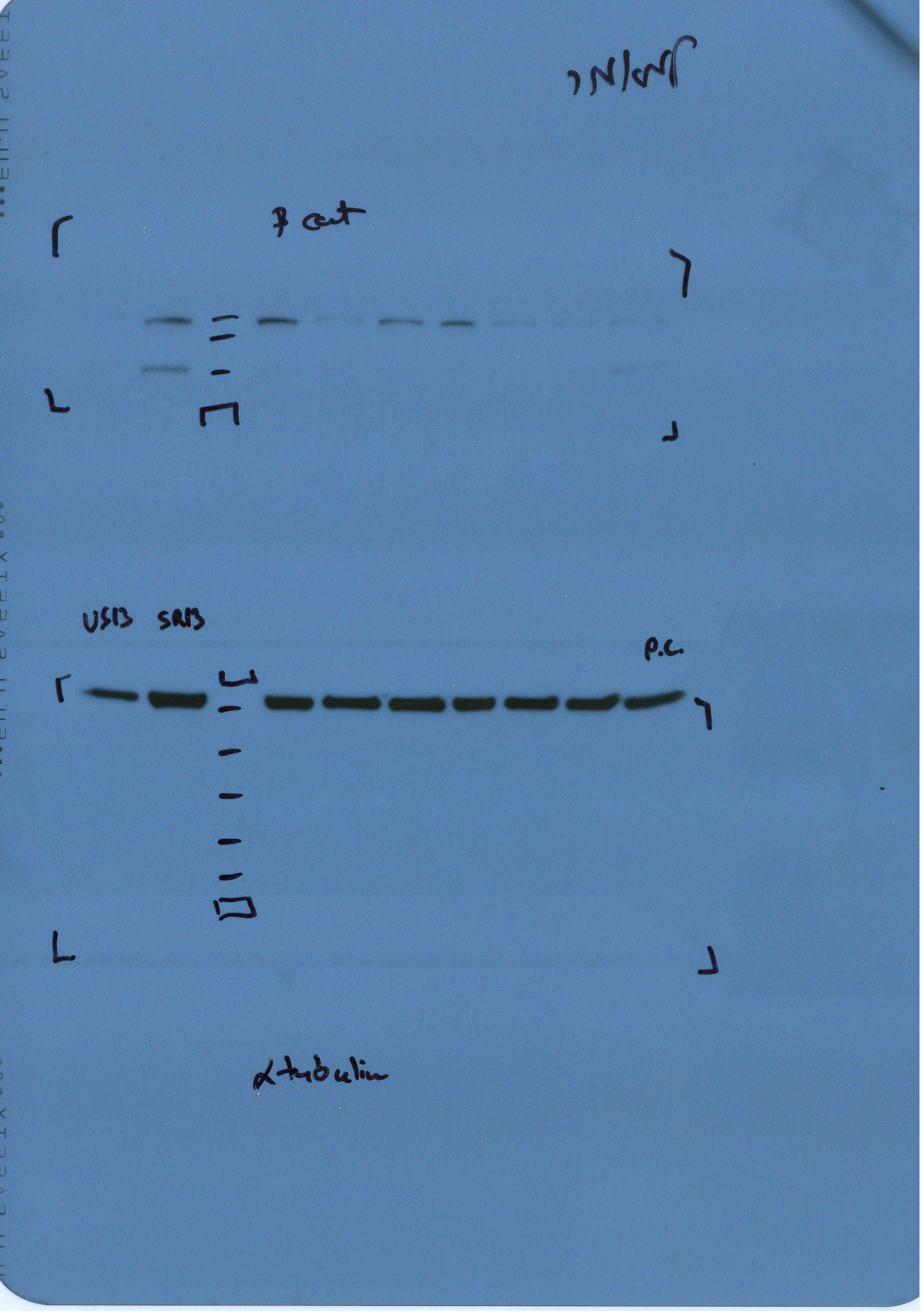

Supplement: Supplementary file 12 [file Image12.tif]

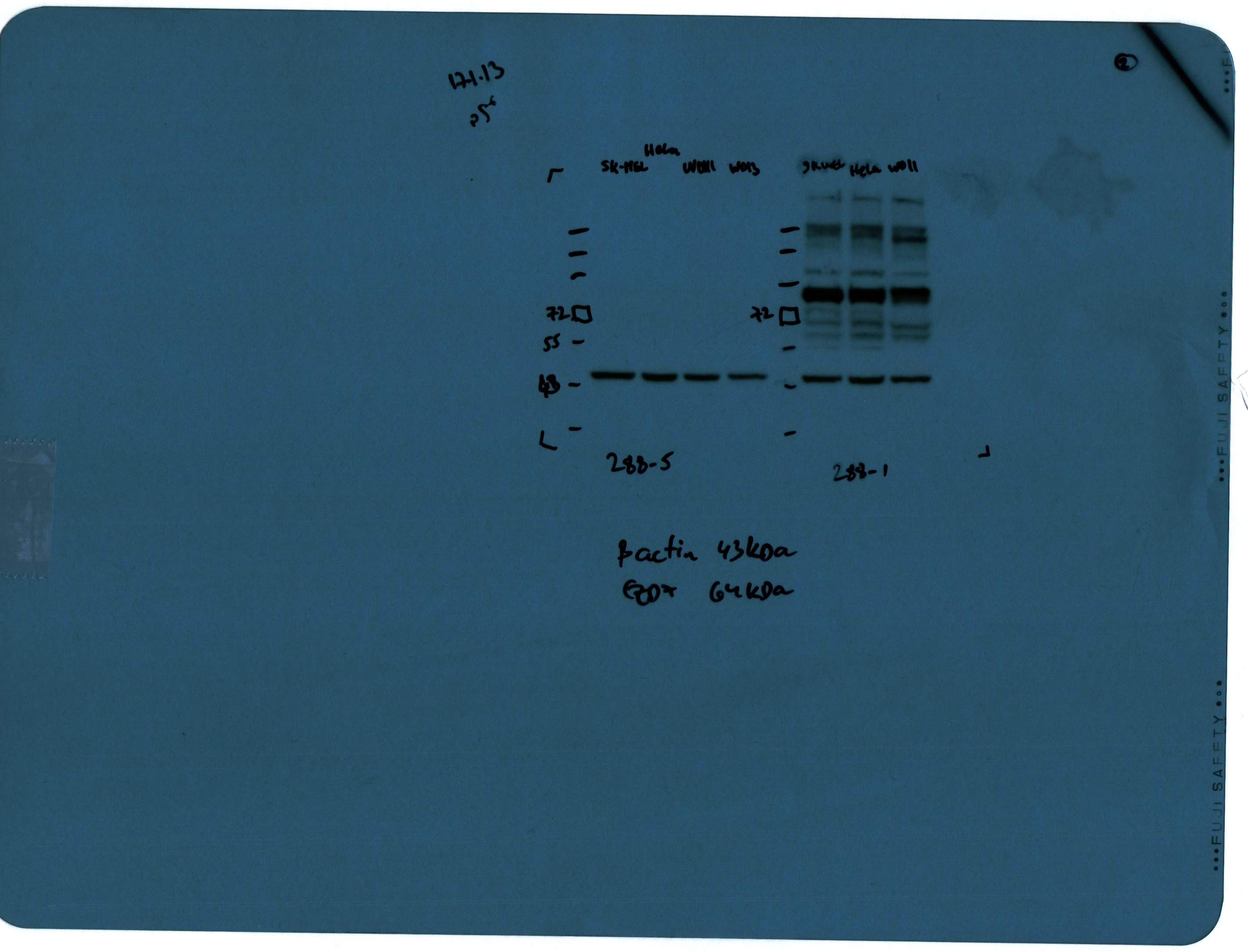

Supplement: Supplementary file 13 [file Image7.tiff]
