# Supplementary material for: A novel Frizzled 7 antibody disrupts the Wnt pathway and inhibits Wilms tumor growth
Source: Front Bioeng Biotechnol. 2025 Sep 24;13:1641137. doi: 10.3389/fbioe.2025.1641137 (PMC12504202; doi:10.3389/fbioe.2025.1641137)
Supplement: Supplementary file 2 [file Supplementaryfile1.docx]

**Supplementary material**

**Tables:**

Supplementary Table S1: Stable clones matching to immunizing peptides

Supplementary Table S2: Tumor volume measured before the treatment

Supplementary Table S3: Wilms' Tumor patients characteristics

Supplementary Table S4: Antibodies used for Western blot analysis

Supplementary Table S5: FZD7-shRNA sequences

Supplementary Table S6: TaqMan primer ID for quantitative RT-PCR

Supplementary Table S7: Primary and secondary IF antibodies

**Figures:**

Supplementary Figure S1: Representative flow cytometry analysis of FZD7 expression in SK-MEL28 cells

Supplementary Figure S2: SK-MEL28 cell lysate tested with commercial and αFZD7-288.1

Supplementary Figure S3: Binding of Immunizing peptide 4 to αFZD7-288.1 and blocking of the Ab-receptor interaction

Supplementary Figure S4: Relative growth ratios of cells treated with the specific αFZD7-288.1 Ab in varying concentrations

Supplementary Figure S5: Real-time PCR gene expression analysis of canonical Wnt pathway genes in SK-MEL28 cells

Supplementary Figure S6: Canonical Wnt inhibition in SK-MEL28 cells

Supplementary Figure S7: Canonical Wnt inhibition in HeLa cells

Supplementary Figure S8: Inhibition of Wnt signaling by αFZD7-288.1 in high volume tumors decreased tumor growth

**Supplementary material**

**Table S1: Stable clones matching to immunizing peptides**

| **Clone** | **Peptide**  **(verified by ELISA)** |
| --- | --- |
| 288.1 | 4 |
| 288.2 | 4 |
| 288.5 | 4 |
| 289.18 | 3 |

**Table S2: Tumor volume measured before treatment**

| **Tumor volume (mm3)** | |
| --- | --- |
| **Saline** | |
| 1 | 328.7 |
| 2 | 436.3 |
| 3 | 430.6 |
| 4 | 415.4 |
| 5 | 341.5 |
| 6 | 387.1 |
| **Avg** | **389.9** |
| **SEM** | **18.8** |
| **αFZD7-288.1** | |
| 1 | 293.3 |
| 2 | 413.3 |
| 3 | 371.1 |
| 4 | 373.4 |
| 5 | 367.6 |
| **Avg** | **363.7** |
| **SEM** | **17.8** |
| **PTX** | |
| 1 | 138.5 |
| 2 | 339.5 |
| 3 | 450.5 |
| **Avg** | **309.5** |
| **SEM** | **79.1** |

**Table S3: Wilms' Tumor patients characteristics**

| **Patient Code** | **Gender** | **Age** | **Pattern** | **Histology** | **Remarks** |
| --- | --- | --- | --- | --- | --- |
| W002 | Female | 4 years | Triphasic | Favorable histology | Lung metastasis |
| W005 | Male | 3 years | Triphasic | Favorable histology | - |
| W009 | Male | 3 years | Triphasic | Unfavorable histology | Recurrent with diffused anaplasia |
| W011 | Female | 7 years | Triphasic - Blastemal predominance | Favorable histology | Recurrent WT with Liver metastasis |
| W013 | Male | 4 years | Triphasic | Favorable histology | - |
| W014 | Male | 9 months | Triphasic | Favorable histology | - |
| W016 | Female | 2 rears | Triphasic | Favorable histology | - |
| W026 | Male | 3 years | Triphasic | Favorable histology | - |
| W027 | Male | 1 year | Triphasic | Favorable histology | - |
| W030 | Female | 11 months | Triphasic | Favorable histology | - |
| W038 | Male | 5 years | Blastemal predominance | Favorable histology | - |
| W041 | Male | 4 years | Biphasic | Favorable histology | - |

**Table S4: Antibodies used for Western blot analysis**

| **WB Antibody** | **Host** | **Size** | **Dilution** | **Manufacturer** | **Cat No.** |
| --- | --- | --- | --- | --- | --- |
| Active β-catenin | mouse | 92KDa | 1:500 | Millipore | 05-665 |
| FZD7 | rabbit | 64Kda | 1:1000 | Millipore | 06-1063 |
| α-Tubulin | mouse | 55KDa | 1:10000 | Epitomics | 1878-1 |
| β-actin | rabbit | 43KDa | 1:20000 | Epitomics | 1844-1 |
| 2’ Peroxidase-AffiniPure Goat Anti-Mouse IgG | - | - | 1:10000 | Jackson ImmunoResearch | 115-035-146 |
| 2’ Peroxidase-AffiniPure Goat Anti-Rabbit IgG | - | - | 1:10000 | Jackson ImmunoResearch | 111-035-144 |

**Table S5: FZD7-shRNA sequences**

**Table S6: TaqMan primer ID for quantitative RT-PCR**

**Table S7: Primary and secondary IF antibodies**

| **Antibodies** |  | **Host** | **Manufacturer** | **Cat No.** |
| --- | --- | --- | --- | --- |
| β-catenin | IF | mouse | Millipore | MAB2081 |
| Cytokeratin | IF | rabbit | Dako | Z0622 |
| FZD7-288.1 | IF | mouse | Sigma | - |
| Ki67  NCAM1 | IF  IF | Mouse  Rabbit | Vector  Proteintech | VP-K452  14255-1-AP |
| Anti-Mouse - alexa 555 | IF | donkey | Life technologies | A31570 |
| Anti-Rabbit - alexa 488 | IF | donkey | Life technologies | A21206 |
| Ki67 | IHC | rabbit | Thermofisher Scientific | RM9106-R7 (clone SP6) |
| Caspase3 | IHC | rabbit | Abcam | Ab4051 |

**Figure S1: Representative flow cytometry analysis of FZD7 expression in SK-MEL28 cells**


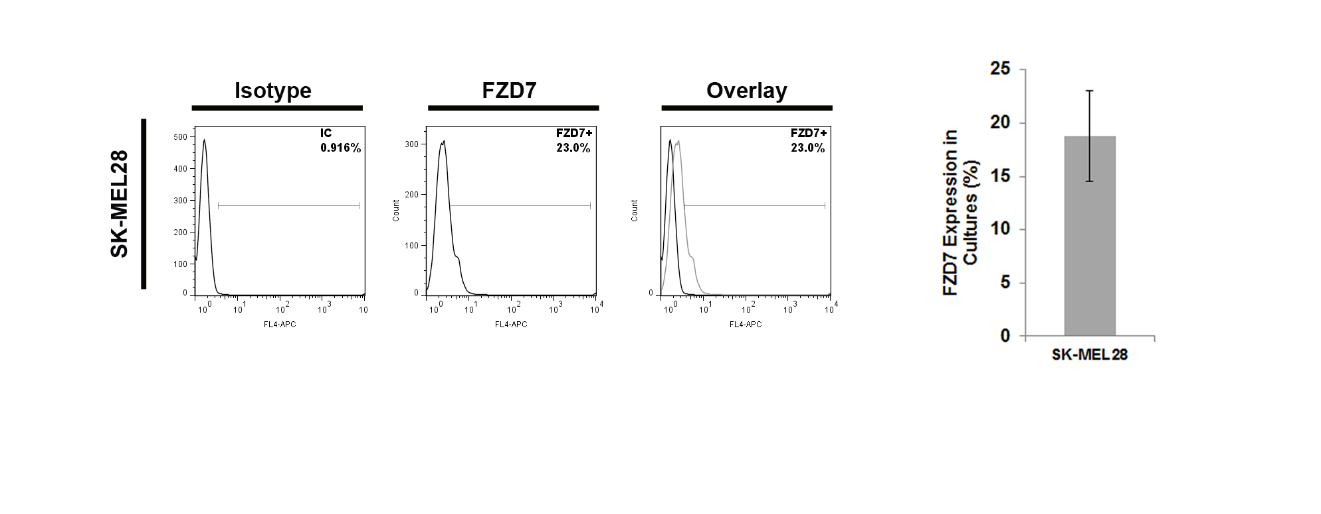


Representative flow cytometry analysis of FZD7 expression in SK-MEL28 cells and summarizing bar graph of average FZD7 expression in SK-MEL28 cells used for the *in-vitro* experiments. Results are shown as mean±SEM, representing at least 3 different FZD7 level analyses.

**
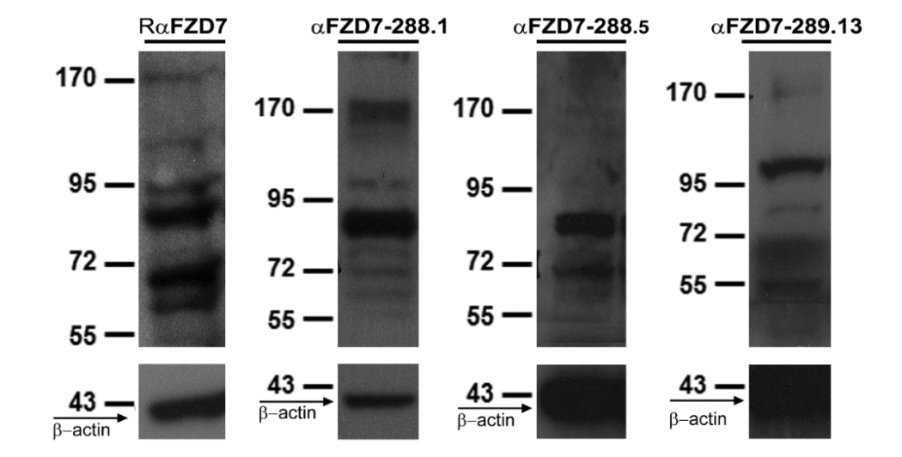
**

**Figure S2: SK-MEL28 cell lysate tested with commercial and αFZD7-288.1**

SK-MEL28 cell lysate was separated by 10% SDS-PAGE gel and probed using the indicated anti-FZD7 antibodies. Results show similar band sizes identified by the different Abs, including the predicted 64KDa protein size as well as a slightly higher band due to phosphorylation, and two heavier bands at 85-95KDa, possibly due to protein glycosylation, and a combination of both modifications. Doublets of proteins are also identified at 130KDa for the unmodified FZD7 and at about 170KDa for the modified protein. β-actin was used as loading control.

**
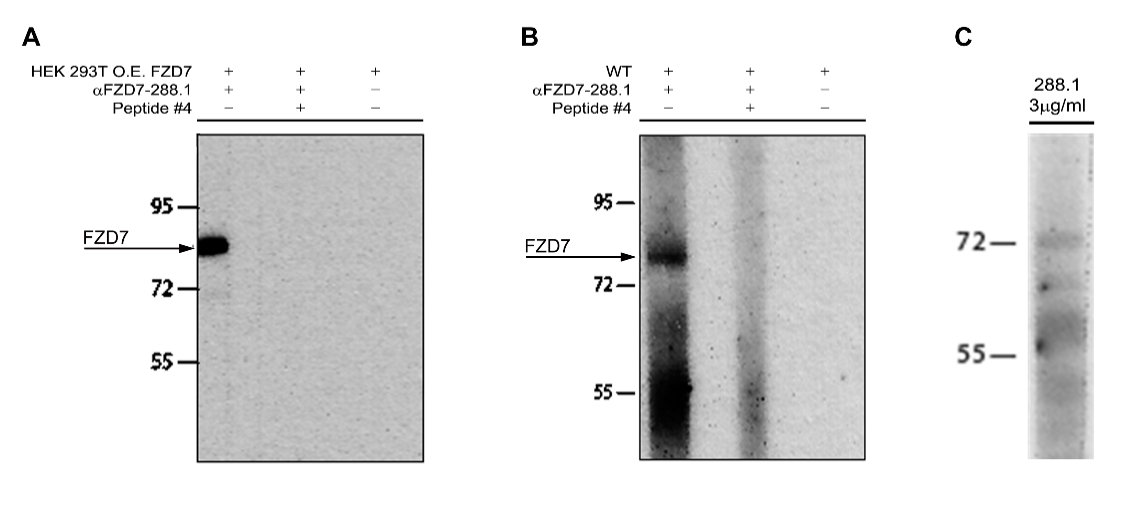
Figure S3: Binding of Immunizing peptide 4 to αFZD7-288.1 and blocking of the Ab-receptor interaction**

Immunizing peptide 4 binds to αFZD7-288.1 and blocks the Ab-receptor interaction. αFZD7-288.1 was incubated with the immunizing peptide for 4h. HEK293T over-expressing FZD7 **(A)** and WT **(B)** cell lysate were assayed by Western Blot. FZD7 protein detection was done by either αFZD7-288.1 (left lane), blocked αFZD7-288.1 (middle lane), or negative control (right lane).

**
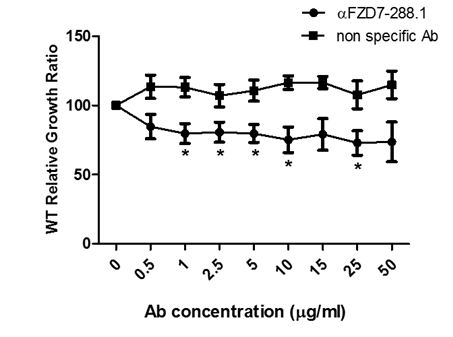
Figure S4: Relative growth ratios of cells treated with the specific αFZD7-288.1 Ab in varying concentrations**

Relative growth ratios of cells treated with the specific αFZD7-288.1 Ab in varying concentrations, compared to a nonspecific Ab. Proliferation of untreated cells set as 100%; Results shown as mean±SEM, representing at least 3 different experiments (*p<0.05).

**Figure S5: Real-time PCR gene expression analysis of canonical Wnt pathway genes in SK-MEL28 cells**

**
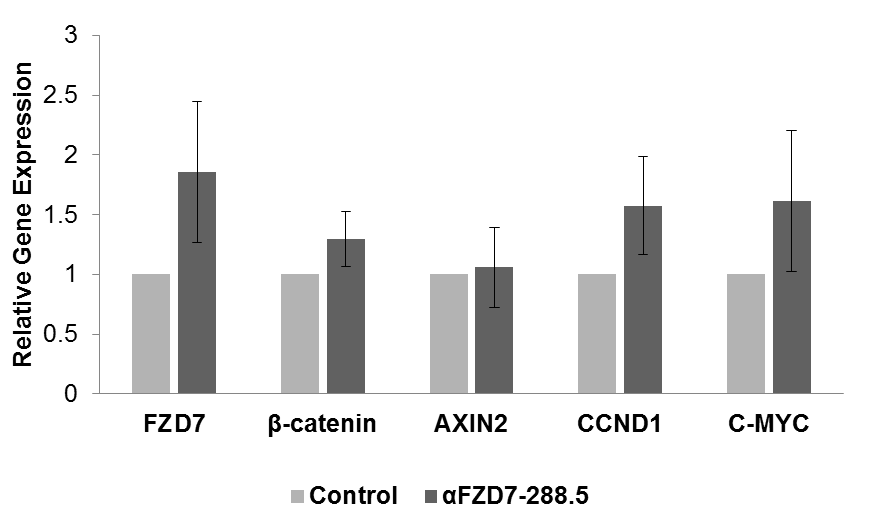
**

Real-time PCR gene expression analysis of canonical Wnt pathway genes in SK-MEL28 cells (Data shown as mean ± S.E.M of 3 separate experiments).

**
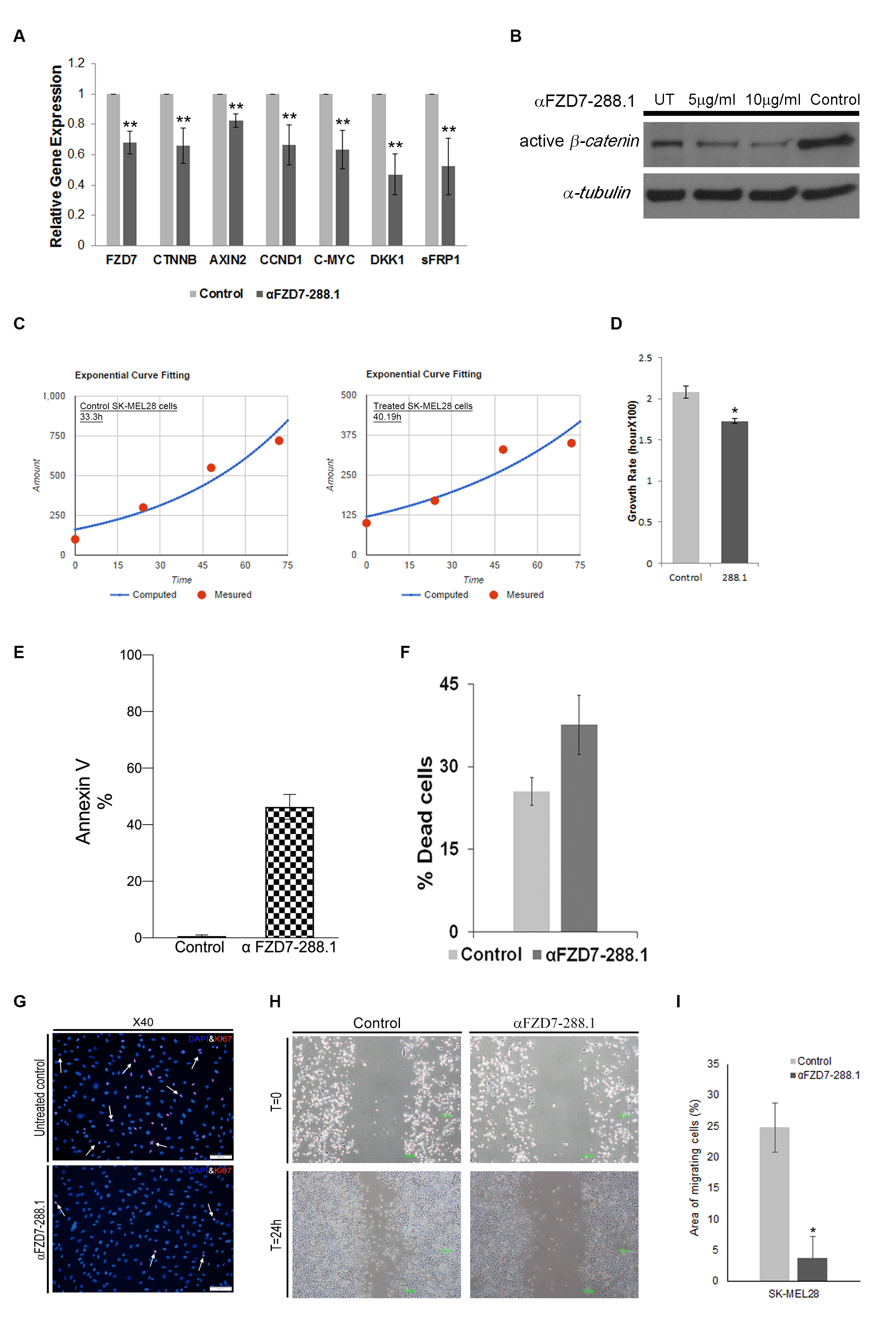
**

**Figure S6: Canonical Wnt inhibition in SK-MEL28 cells**

Canonical Wnt inhibition in SK-MEL28 cells: **(A)** Real-time PCR gene expression analysis of Wnt pathway related genes showed reduced mRNA expression in SK-MEL28 cells (Data shown as mean ± S.E.M of 5 separate experiments, *p<0.05; **p<0.01). **(B)** Western blot analysis demonstrating inhibition of active β-catenin following treatment with 5µg/ml or 10µg/ml of αFZD7-288.1 in SK-MEL28 cells. Nonspecific Ab was used as negative control; α-tubulin was used for loading control. Inhibition of the Wnt pathway by αFZD7-288.1 prompted longer doubling time of SK-MEL28 cells compared to control **(C)**, and reduced growth rate of SK-MEL28 cells (**D**; *p<0.05; n=4). αFZD7-288.1 treatment of SK-MEL28 induces cell death as shown by representative dot-plot analysis for annexinV staining **(E)** and by calculation of the percentage of dead cells using trypan blue staining **(F**; *p<0.05; n=4). αFZD7-288.1 inhibits SK-MEL28 cells proliferation as evident by reduced nuclear Ki67 expression (red, lower panels) compared to control untreated cells (**G**; magnification X40 scale bars=100µm; magnification X20 scale bars=50µm). Following αFZD7-288.1 treatment, SK-MEL28 cells demonstrated lower migration capacity compared to control cells, as seen in the representative image **(H)**. Calculation of the area of migrating cells showed significantly reduced migration capacity (**I**;*p<0.05; n=3).

**Figure S7: Canonical Wnt inhibition in HeLa cells**

**
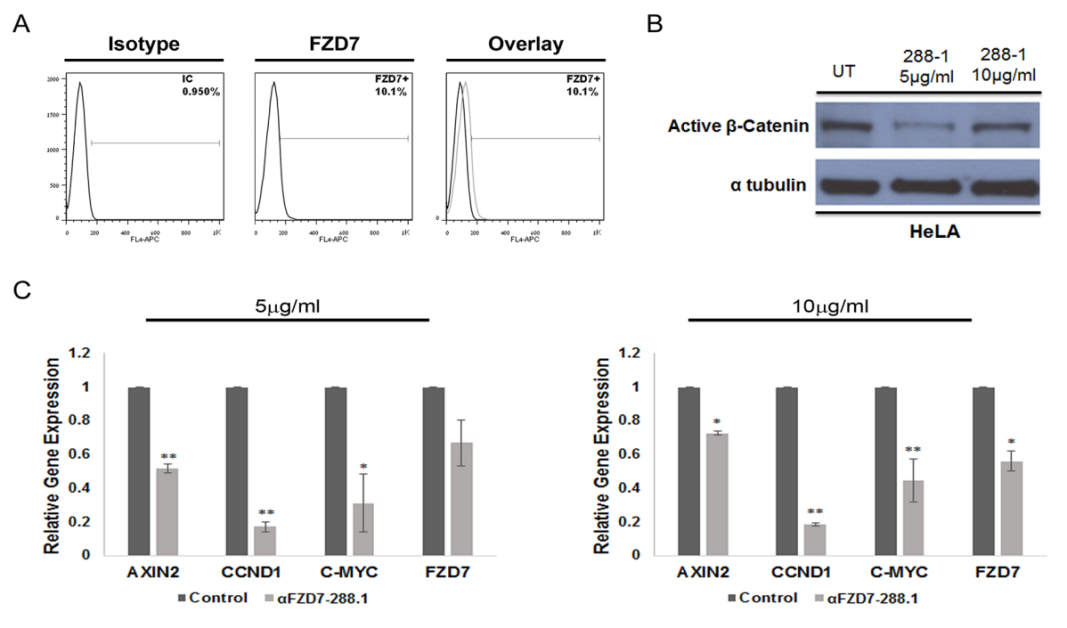
**

Canonical Wnt inhibition in HeLa cells: **(A)** Representative flow cytometry graph with expression of FZD7 in HeLa cells. **(B)** Western blot analysis demonstrating inhibition of active β-catenin following treatment with 5µg/ml or 10µg/ml of αFZD7-288.1 for 48. α-tubulin was used for loading control. **(C)** Real-time PCR gene expression analysis of Wnt pathway target genes (AXIN2, CCND1, MYC-C), and of FZD7. Results showed reduced mRNA expression of Wnt pathway-related genes in HeLa cells (Data shown as mean ± S.E.M of 3 separate experiments, *p<0.05; **p<0.01).

**Figure S8: Inhibition of Wnt signaling by αFZD7-288.1 in high volume tumors decreased tumor growth**


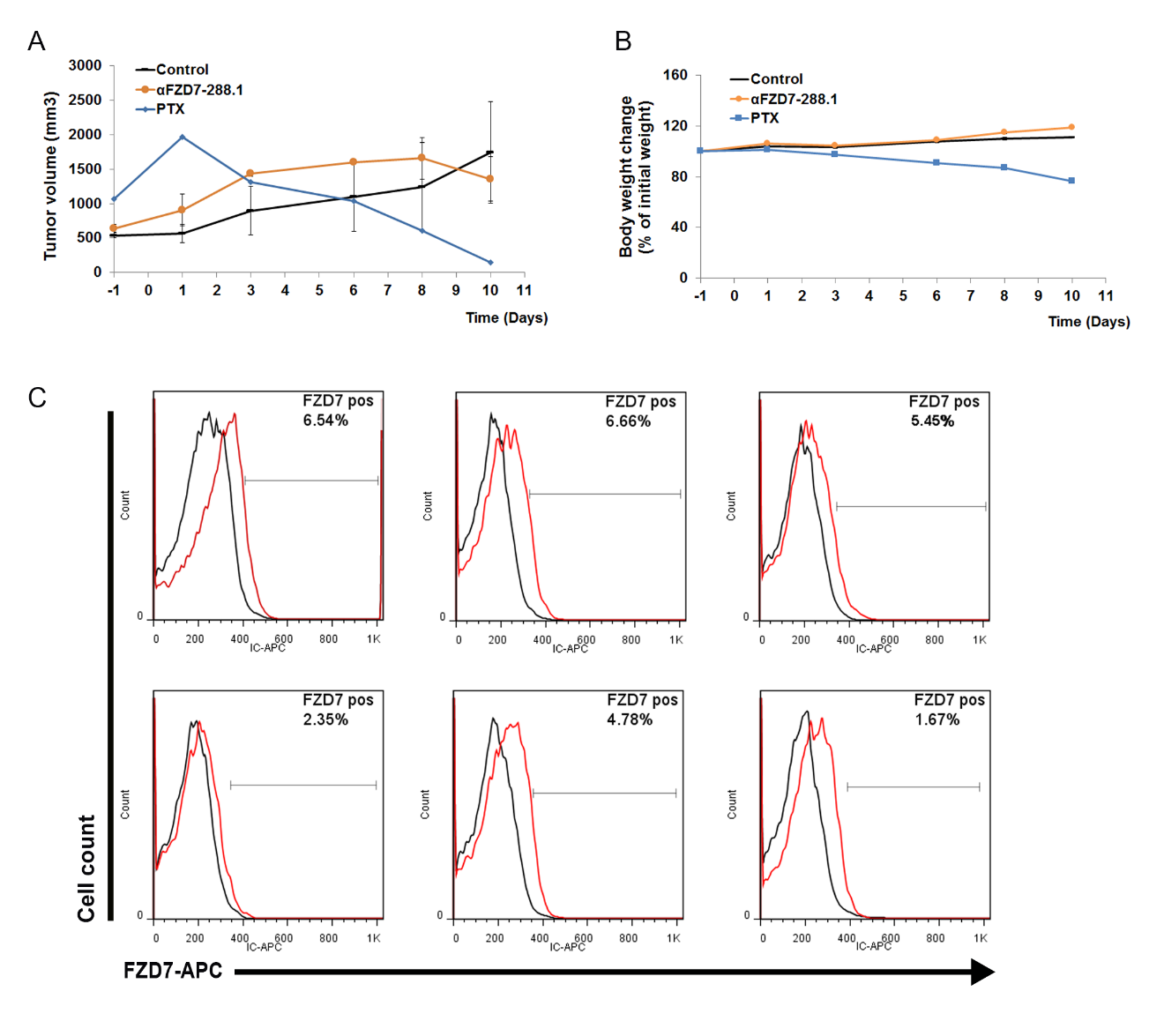


Inhibition of Wnt signaling by αFZD7-288.1 in high volume tumors decreased tumor growth. **(A)** Antitumor activity: Mice were treated as previously described. Tumors treated with αFZD7-288.1 exhibited growth inhibition only after the fifth injection while PTX treated tumors showed antitumor response after the second injection. Tumors treated with saline showed continuous tumor growth. **(B)** Mouse weights during treatment. PTX treated mice suffered from toxic side effect and significant weight loss following treatment (**C**) Flow cytometry analysis of the tumors processed from the single-cell cohort demonstrated a significant decrease (fold change of 0.445) in the percentage of FZD7-expressing cells in the αFZD7-288.1-treated tumors compared to that in the control group
